# Modified Nucleotides for Discrimination between Cytosine and the Epigenetic Marker 5‐Methylcytosine

**DOI:** 10.1002/anie.201511520

**Published:** 2016-02-02

**Authors:** Janina von Watzdorf, Kim Leitner, Andreas Marx

**Affiliations:** ^1^ Fachbereich Chemie, Graduiertenschule Chemische, Biologie KonstanzUniversität KonstanzUniversitätsstrasse 1078457KonstanzDeutschland

**Keywords:** 5-methylcytosine, DNA methylation, DNA polymerase, epigenetics, polymerase chain reaction

## Abstract

5‐Methyl‐2′‐deoxycytosine, the most common epigenetic marker of DNA in eukaryotic cells, plays a key role in gene regulation and affects various cellular processes such as development and carcinogenesis. Therefore, the detection of 5mC can serve as an important biomarker for diagnostics. Here we describe that modified dGTP analogues as well as modified primers are able to sense the presence or absence of a single methylation of C, even though this modification does not interfere directly with Watson–Crick nucleobase pairing. By screening several modified nucleotide scaffolds, O^6^‐modified 2′‐deoxyguanosine analogues were identified as discriminating between C and 5mC. These modified nucleotides might find application in site‐specific 5mC detection, for example, through real‐time PCR approaches.

Epigenetic modifications, caused by methylation of cytosine residues (5‐methyl‐2′‐deoxycytosine, 5mC; Figure 1), have been proven to have an impact on a variety of cellular processes that affect development^1^, ^2^ and gene expression^3^ as well as the development of various diseases.^4^ Genes are frequently found to be silenced^3^ if CpG dinucleotides in the corresponding promoters exhibit significant levels of 5mC; thus, 5mC is known to regulate gene transcription and thereby affect tumorigenesis.^5^ The level of epigenetic methylation has to be precisely regulated in eukaryotic genomes, since changes of the methylation pattern lead to severe genetic malfunctions.^6^

**Figure 1:**
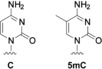
Chemical structure of C (left) and 5mC (right).

Therefore, the detection of the occurrence and distribution of 5mC in the genome holds the potential to serve as an important biomarker for diagnosis as well as disease therapy.^7^ This requires efficient strategies for the detection of 5mC. Different concepts for discriminating between cytosine (C) and 5mC have been described which rely on endonuclease digestion,^8^ affinity enrichment,^9^ nanopore sequencing,^10^ different chemical behavior concerning redox reactivity,^11^ or selective deamination of C using sodium bisulfite.^12^

Bisulfite sequencing has become routine for the detection of 5mC with single‐nucleotide resolution.^13^ This method is based on the selective bisulfite‐mediated deamination of C to uracil (U) in the presence of 5mC, which remains unaffected as a result of slower deamination.^14^ The sites of epigenetic markers can be revealed by comparison of the output of conventional sequencing methods before and after bisulfite treatment, as C will be sequenced as thymine (T), and 5mC as C.^15^

Although bisulfite sequencing can be used for the genome‐wide detection of 5mC, it possesses several drawbacks. Since many steps are required and two sequencing runs are needed for comparison, the method is time consuming and prone to contaminations.^16^ In addition, the conditions used for the bisulfite treatment are harsh and destroy approximately 95 % of the genomic DNA, and thus a large amount of DNA is required.^17^ Furthermore, deamination of C and 5mC after bisulfite treatment is incomplete, thereby leading to an error‐prone output.^18^

Nucleotides that are able to discriminate between C and 5mC would be highly interesting tools for the development of new approaches for the site‐specific detection of 5mC. However, since the methylation of cytosines at C5 does not directly affect Watson–Crick base pairing, attempts along these lines are challenging and have not yet been described. Here we present a class of modified nucleotides that can be used for discrimination between C and 5mC in reactions catalyzed by DNA polymerases. For this purpose, we screened and investigated several purine‐based 2′‐deoxynucleotides for their ability to sense 5mC as a result of diverging incorporation efficiencies opposite a template containing C or 5mC by different DNA polymerases (Figure 2). We found that several *O* ^6^‐modified 2′‐deoxyguanosine derivatives (Figure 3 a) are incorporated opposite 5mC and extended from 5mC with significantly different efficiencies compared to the unmodified counterparts.

First, we screened a variety of different modified nucleotides (Figure 2) in combination with the thermostable DNA polymerases *KlenTaq* and *KOD* exo^− [19]^ in primer extension experiments, followed by analysis through denaturing polyacrylamide gel electrophoresis (PAGE) and visualization by autoradiography. Both DNA polymerases were able to incorporate differently modified nucleotides opposite C or 5mC, although with decreased incorporation efficiencies compared to the unmodified dGTP (**1**; Figure 2). *KOD* exo^−^ showed the highest potency for the desired application, with the most pronounced differences in the incorporation efficiencies being observed during the processing of the nucleotides *O* ^6^‐methyl‐dGTP (**3**), dATP (**10**), and 5‐nitro‐1‐indolyl‐2′‐deoxyribose‐5′‐triphosphate (**21**). Since modified dATP analogues (nucleotides **11**–**16**) showed decreased incorporation efficiencies as well as decreased discrimination between both templates, and the synthesis of derivatives of nucleotide **21** seems to be tedious and challenging, we decided to focus on *O* ^6^‐methyl‐dGTP (**3**) for further derivatization. *KlenTaq* DNA polymerase showed no significant difference in incorporation efficiencies for either dGTP or the modified nucleotides (see Figure S1 in the Supporting Information).

**Figure 2:**
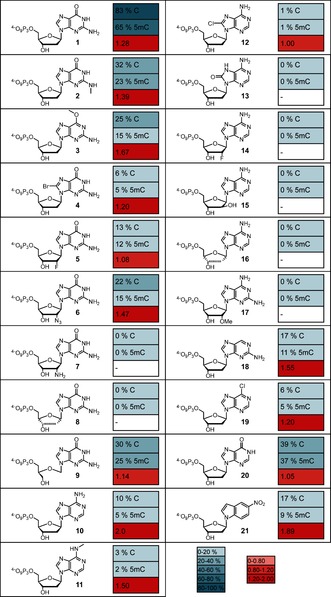
Structures of modified dNTP analogues, including the percentage incorporation opposite C or 5mC on employing *KOD* exo^−^ in primer extension by incorporation of a single nucleotide. 50 μm dGTP or dN*TP and 5 nm *KOD* exo^−^ were used; the reactions were stopped after 20 min. Discrimination ratios were determined by calculating the quotient of the % incorporation opposite C and % incorporation opposite 5mC.

Next, we focused on optimizing the system by synthesis and functional evaluation of *O* ^6^‐alkylated dGTPs. *O* ^6^‐Alkylated dGTP derivatives were synthesized starting from commercially available 2′‐deoxyguanosine, which was acetylated and subsequently chlorinated at the 6‐position by known procedures (see Figure S2 in the Supporting Information).^20^ The different alkoxy groups were introduced in position 6 by the reaction of **22** with the respective sodium alkoxide solutions. The obtained nucleosides **23 a**–**c** were converted into the corresponding 2′‐deoxynucleoside‐5′‐triphosphates (Figure S2).^21^, ^22^

The potency of *O* ^6^‐methyl‐, *O* ^6^‐ethyl‐, *O* ^6^‐propyl‐, and *O* ^6^‐isopropyl‐dGTP towards diverging incorporation efficiencies opposite C and 5mC was further investigated. Both DNA polymerases were able to incorporate all four nucleotide analogues opposite C and 5mC, although the incorporation efficiency decreased with increased steric demand of the introduced modifications (Figure 3 b and see Scheme S4). *KOD* exo^−^ showed the highest potency for the desired application, with the most pronounced differences in incorporation efficiencies being observed during the processing of the nucleotides dGTP, *O* ^6^‐methyl‐, *O* ^6^‐ethyl‐, *O* ^6^‐propyl‐, and *O* ^6^‐isopropyl‐dGTP opposite C compared to 5mC (Figure 3 b). Although the processing efficiencies of those modified nucleotides were decreased compared to natural dGTP, the discrimination between the two templates was increased markedly (Figure 3 b). Of note, as reported before, *O* ^6^‐methyl‐dGTP was also processed opposite T^23^ and we could show that the same holds true for the other *O* ^6^‐alkyl‐dGTP derivatives (Figure S5). In contrast, *KlenTaq* DNA polymerase again showed no significant difference in incorporation efficiencies for either dGTP or the modified nucleotides (Figure S4).

**Figure 3:**
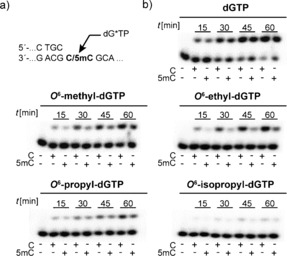
a) Partial primer/template sequence used. b) PAGE analysis of primer extension experiments on incorporation of a single nucleotide of dGTP, *O* ^6^‐methyl‐, *O* ^6^‐ethyl‐, *O* ^6^‐propyl‐, and *O* ^6^‐isopropyl‐dGTP opposite a template containing C, in comparison to a template containing 5mC (with use of *KOD* exo^−^). 50 μm dGTP or dG*TP and 5 nm *KOD* exo^−^ were used; reactions were stopped after the indicated time points.

To further investigate this observation, we determined the steady‐state kinetics^24^ for the processing of the nucleotides dGTP, *O* ^6^‐methyl‐, *O* ^6^‐ethyl‐, *O* ^6^‐propyl‐, and *O* ^6^‐isopropyl‐dGTP by *KOD* exo^−^ opposite C and 5mC (Table 1; see also Scheme S2 and Figure S7). Comparison of the catalytic efficiencies (*k*_cat_/*K*_m_) observed during processing of dGTP and the modified nucleotides opposite C or 5mC in the template strand confirms all the tendencies observed in the qualitative primer extension reactions (Table 1 and see Scheme S2). The ratio of the catalytic efficiency observed during incorporation of the respective nucleotide opposite C compared to 5mC varied. The best discrimination was observed for the processing of *O* ^6^‐ethyl‐dGTP, with the catalytic efficiencies *k*_cat_/*K*_m_ for incorporation opposite C and 5mC differing by a factor of 4.2 (Table 1 and see Scheme S2).

**Table 1:** Steady‐state kinetic analysis of primer extension on incorporation of a single nucleotide opposite C or 5mC by *KOD* exo^−^ DNA polymerase.^[a]^

(3′‐)Nucleotide	Template	*K*_cat_/*K*_m_ [s^−1^ μm ^−1^]	Ratio
dGTP	C	1.5±0.1	1.36
	5mC	1.1±0.6	
*O* ^6^‐methyl‐dGTP	C	0.16±0.02	2.58
	5mC	0.062±0.010	
*O* ^6^‐ethyl‐dGTP	C	0.14±0.02	4.24
	5mC	0.033±0.007	
*O* ^6^‐propyl‐dGTP	C	0.105±0.015	1.08
	5mC	0.097±0.011	
*O* ^6^‐ isopropyl‐dGTP	C	0.031±0.005	1.48
	5mC	0.021±0.004	
G	C	8.7±1.3	3.48
	5mC	30.3±3.4	
*O* ^6^‐methyl‐G	C	0.0043±0.0008	6.72
	5mC	0.0289±0.0079	
*O* ^6^‐ethyl‐G	C	0.0066±0.0002	3.53
	5mC	0.0233±0.0080	

[a] Ratio: *k*_cat_/*K*_m_ (C) to *k*_cat_/*K*_m_ (5mC) (nucleotide incorporation) or *k*_cat_/*K*_m_ (5mC) to *k*_cat_/*K*_m_ (C) (modified primer extension).

Next, we aimed at investigating the effect of the modifications when they are embedded at the 3′‐terminus of primer strands and placed directly opposite C or 5mC.^25^ Thus, the modified nucleosides were converted into phosphoramidites, which were employed in DNA solid‐phase synthesis of the different primer strands (see Figure S3 in the Supporting Information). The methyl and ethyl modifications in *O* ^6^‐methyl‐dGTP and *O* ^6^‐ethyl‐dGTP showed the most promising results, and so we focused on these modifications (Figure 3, Table S1).

After their synthesis, the primer strands were first employed in primer extension studies (Figure 4 b). Again, both modifications were well tolerated by both DNA polymerases tested, with all three primers elongated, although the modified primers to a smaller extent than the unmodified primer (Figure 4 b and see Figure S6). Interestingly, no significant discrimination between C and 5mC was observed with *KOD* exo^−^, either when the unmodified or when the modified primer strands were employed (Figure S6). In contrast, significant discrimination was observed with the *KlenTaq* DNA polymerase for both modified primers (Figure 4 b). A marginal difference in the incorporation efficiencies between extension from C or 5mC was observed when the unmodified primer was extended with dCTP. In contrast, elongation of the modified primers bearing either *O* ^6^‐methyl‐G or *O* ^6^‐ethyl‐G at the 3′‐end showed extensive discrimination between both templates (Figure 4 b).

**Figure 4:**
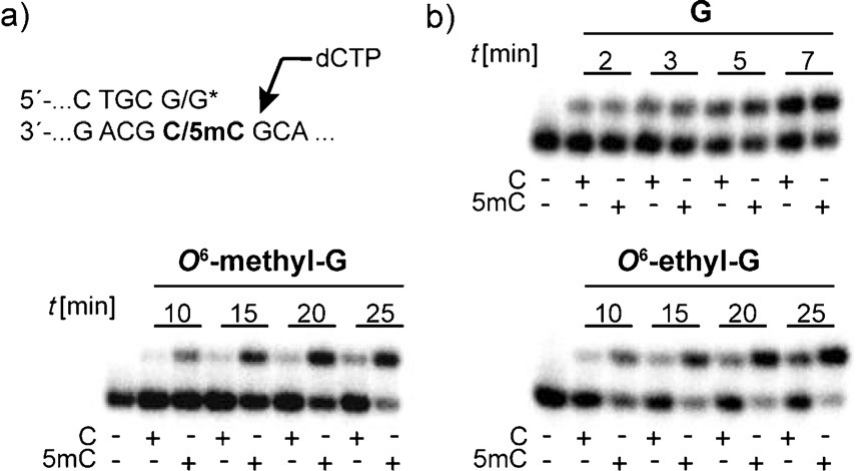
a) Sequence context of the primer/template complex used in the described studies. b) PAGE analysis of the primer extension experiments for incorporation after C or 5mC paired with the 3′‐terminally unmodified G or modified *O* ^6^‐methyl‐G and *O* ^6^‐ethyl‐G primers (by using *KlenTaq*). 50 μm dCTP and 0.1 nm *KlenTaq* were used for G, 10 nm *KlenTaq* was used for *O* ^6^‐methyl‐G and *O* ^6^‐ethyl‐G; reactions were stopped after the indicated time points.

Interestingly, in these primer extension reactions, elongation from the modified primer is more efficient when paired with 5mC. To confirm these results, we determined steady‐state kinetics^24^ using *KlenTaq* DNA polymerase in combination with the unmodified primer and the two modified primers paired with both templates (Table S3, Figure S8). These experiments confirm that both modified primers are elongated with lower efficiency than the unmodified primer and that elongation of the primers from 5mC is more efficient. We found that the primer bearing *O* ^6^‐methyl‐G showed the best discrimination between C and 5mC (Table 1 and Scheme S3). The catalytic efficiencies (*k*_cat_/*K*_m_) for the two reactions differ by a factor of 6.7 (Table 1).

The different behavior of the DNA polymerases *KlenTaq* and *KOD* exo^−^ when processing the modified nucleotides in the context of C and 5mC is intriguing. Structural data of both enzymes bound to a primer and a template reveals significantly different interaction patterns of the respective protein with its substrate.^26^ This difference might be the origin of the observed effects.

Next we investigated whether we were able to exploit these findings in polymerase chain reaction (PCR) experiments. We used *KlenTaq* DNA polymerase and a primer modified at the 3′‐terminus. The modified nucleotide forms a base pair with a methylated or unmethylated cytosine in human genomic DNA (gDNA; Figure 5). We designed a forward primer that should lead to a 155 bp PCR product and analyzed a specific CpG site in the promotor region of NANOG in gDNA.^1^, ^27^ We used gDNA purified from HeLa cells because it was shown that this CpG site is unmethylated.27b However, an enzymatically methylated HeLa gDNA was used as a fully methylated control. We found in real‐time PCR experiments that discrimination between both un‐ and methylated cytosine in gDNA was improved by using the primer bearing *O* ^6^‐methyl‐G at the 3′‐end relative to the unmodified primer (Figure 5). The use of the HeLa gDNA leads to a delayed amplification of 2.95±0.80 (mean±standard deviation (SD), *n*=5) compared to the fully methylated gDNA when paired with the primer modified at the 3′‐terminus. In contrast, the employment of the unmodified primer leads to a delayed amplification of 0.8±0.23 (mean ±SD, *n*=5) in the case of the wild‐type HeLa gDNA (Figure 5).

**Figure 5:**
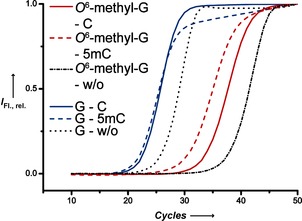
a) Real‐time PCR employing the unmodified (blue) and the 3′‐terminally modified (red) primer from C (solid) and 5mC (dashed) in human HeLa gDNA catalyzed by *KlenTaq* DNA polymerase. PCR curves represent multiple experiments.

In summary, we found that *O* ^6^‐modified 2′‐deoxyguanosine analogues are able to sense the presence or absence of a single methylation in C in the template strand, despite this modification not interfering directly with Watson–Crick nucleobase pairing. Remarkably, opposing trends were observed when the modified nucleotides were incorporated and extension from the modified nucleotides studied. While in the first case *KOD* exo^−^ DNA polymerase discriminates best between C and 5mC by efficient incorporation opposite C, in the latter case *KlenTaq* DNA polymerase discriminates between both cytosines by more efficient extension from 5mC.

We were further able to extend our findings to real‐time PCR‐based systems for the analysis of the methylation status at a single C residue in human gDNA. These results show the potential of using modified nucleotides for application in site‐specific 5mC detection by real‐time PCR, which can be run in parallel for higher throughput without the need for bisulfite treatment. Future investigations will aim at engineering DNA polymerases in combination with modified nucleotides to achieve higher selectivity and elucidate the underlying mechanism in greater detail.

## Supporting information

As a service to our authors and readers, this journal provides supporting information supplied by the authors. Such materials are peer reviewed and may be re‐organized for online delivery, but are not copy‐edited or typeset. Technical support issues arising from supporting information (other than missing files) should be addressed to the authors.

Supplementary
