# Supplementary material for: Modified Nucleotides for Discrimination between Cytosine and the Epigenetic Marker 5‐Methylcytosine
Source: Angew Chem Int Ed Engl. 2016 Feb 2;55(9):3229–32. doi: 10.1002/anie.201511520 (PMC4949677; doi:10.1002/anie.201511520)
Supplement: Supplementary file 1 — Supplementary [file ANIE-55-3229-s001.pdf]

Angewandte Chemie
Eine Zeitschrift der Gesellschaft Deutscher Chemiker

Supporting Information

Modified Nucleotides for Discrimination between Cytosine and the Epigenetic Marker 5-Methylcytosine

*Janina von Watzdorf[+], Kim Leitner[+], and Andreas Marx**

anie_201511520_sm_miscellaneous_information.pdf

SUPPORTING INFORMATION

Index:

Screening (Figure **S1**) S3

Synthesis of 2´-deoxy-5´-triphosphates (Figure **S2**) S4

3´, 5´-Di-*O*-acetyl-2´-deoxyguanosine S5

3´, 5´-Di-*O*-acetyl-6-deoxy-6-chloro-2´-deoxyguanosine (**22**) S5

Alkylation at position 6: General procedure A S5

2´-Deoxy-*O*^{6}-ethyl-guanosine (**23 a**) S6

2´-Deoxy-*O*^{6}-propyl-guanosine (**23 b**) S6

2´-Deoxy-*O*^{6}-*iso*-propyl-guanosine (**23 c**) S6

Triphosphorylation: General procedure B S6

2´-Deoxy-*O*^{6}-ethyl-guanosine-5´-*O*-triphosphate (*O*^{6}-ethyl-dGTP) (**24 a**) S7

2´-Deoxy-*O*^{6}-propyl-guanosine-5´-*O*-triphosphate (*O*^{6}-propyl-dGTP) (**24 b**) S7

2´-Deoxy-*O*^{6}-*iso*-propyl-guanosine-5´-*O*-triphosphate (*O*^{6}-*iso*-propyl-dGTP) (**24 c**) S7

Synthesis of 3´-terminally modified primers S9

Synthesis scheme (Figure **S3**) S9

Iso-butyryl protection of *N*^{2}: General procedure C S9

2´-Deoxy-*N*^{2}-*iso*-butyryl-*O*^{6}-methyl-guanosine (**25 d**) S10

2´-Deoxy-*N*^{2}-*iso*-butyryl-*O*^{6}-ethyl-guanosine (**25 a**) S10

DMTr protection of 5´-OH: General procedure D S10

2´-Deoxy-5´-*O*-(4,4´,-dimethoxytrityl)-*N*^{2}-*iso*-butyryl-*O*^{6}-methyl-guanosine (**26 d**) S10

2´-Deoxy-5´-*O*-(4,4´,-dimethoxytrityl)-N^2-*iso*-butyryl-O^6-ethyl-guanosine (**26 a**) S11

Conversion to phosphoramidite: General procedure E S11

2´-Deoxy-5´-*O*-(4,4´,-dimethoxytrityl)-N^2-*iso*-butyryl-O^6-methyl-guanosine-3´-(2-cyanoethyl-di-*iso*-propyl-phosphoramidite) (**27 d**) S11

2´-Deoxy-5´-*O*-(4,4´,-dimethoxytrityl)-N^2-*iso*-butyryl-O^6-ethyl-guanosine-3´-(2-cyanoethyl-di-*iso*-propyl-phosphoramidite) (**27 a**) S11

Oligonucleotide synthesis S13

Mass and yields of synthesized oligonucleotides (Table **S1**) S13

Biochemical methods S14

Quantification of oligonucleotides S14

5´-Radioactive labelling of oligonucleotides S14

Primer extension assay S14

Gel electrophoresis S15

PAGE analysis of single-nucleotide incorporation PEx of dGTP and O^6-alkyl-dGTP derivatives opposite C or 5mC employing *KlenTaq* (Figure **S4**) S16

PAGE analysis of single-nucleotide incorporation PEx of dGTP and O^6-alkyl-dGTP derivatives opposite C, T, G and A employing *KOD exo*$^-$ (Figure **S5**) S17

PAGE analysis of single-nucleotide incorporation PEx of primers bearing G, O^6-methyl-G or O^6-ethyl-G at the 3'-end opposite C or 5mC employing *KOD exo*$^-$ (Figure **S6**) S18

Enzyme kinetics S19

Steady-state kinetic analysis of dGTP and O^6-alkyl-dGTP derivatives incorporation oppsite C or 5mC employing *KOD exo*$^-$ (Table **S2**; Figure **S7**) S19

Steady-state kinetic analysis of dCTP incorporation in extension of primers bearing G, O^6-methyl-G or O^6-ethyl-G at the 3´-terminus paired with C or 5mC employing *KlenTaq* (Table **S3**; Figure **S8**) S21

PCR experiments S23

NMR spectra S24 – 32

HR-MS spectra of modified primers S34

References S35

Screening:

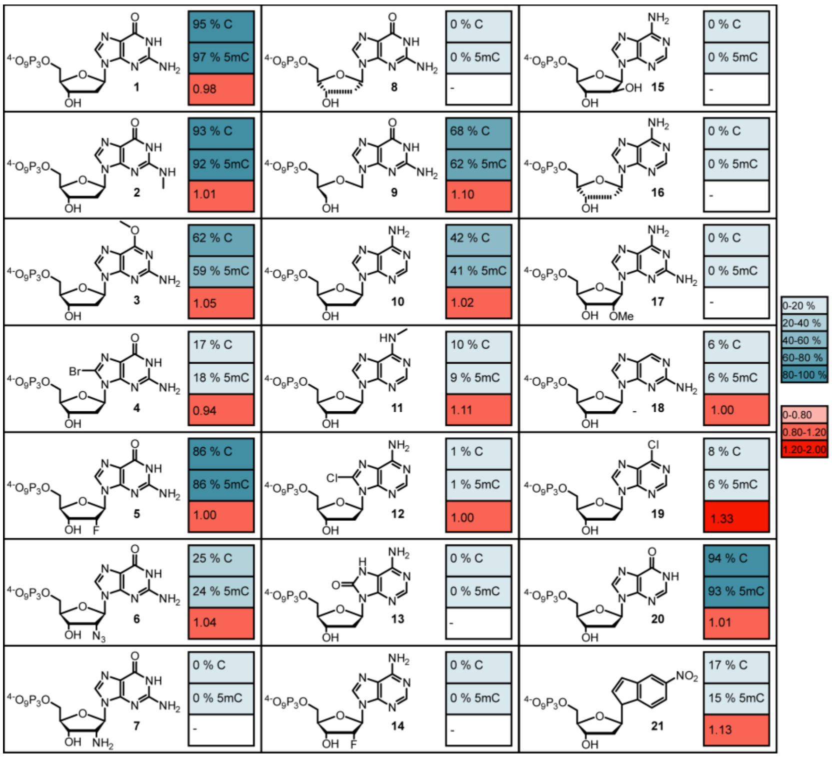

Figure S1. Structures of modified dNTP analogues, including % incorporation opposite C or 5mC employing *KlenTaq* in single-nucleotide incorporation primer extension experiments. 50 µM dGTP or dN*TP and 10 nM *KlenTaq* were used, reactions were stopped after 10 min. Discrimination ratios were determined by calculating the quotient of % incorporation opposite C and % incorporation opposite 5mC.

Synthesis of 2´-deoxy-5´-triphosphates:

Experimental part:

Solvents and reagents were purchased from *Sigma-Aldrich, Fluka, Acros* or *Carbosynth* and were used without further purification. The modified nucleotides were purchased from TriLink Technologies. Dry solvents were purchased from *Sigma-Aldrich*. Mixtures of solvents are given as percent by volume [v/v]. DNA synthesis columns were purchased from ABI Applied Biosystems. DNA polymerases *KlenTaq* and *KOD exo*$^-$ were expressed and purified as described before.[1] T4 polynucleotide kinase PNK was purchased from New England Biolabs. [γ-^{32}P]-ATP was purchased from Hartmann Analytics and natural dNTPs from Roche. Reactions were conducted with exclusion of air and moisture as needed. Thin layer chromatography (TLC) was performed using silica gel 60 F254 aluminium plates from *Merck.* Spots were visualized under UV-light or by staining. Preparative flash chromatography was carried out using silica gel G 60 (40-63 µm, *Merck*) with a pressure of 0.3 bar. NMR spectra were recorded on a Bruker Avance III 400 MHz spectrometer and a Bruker AVIII 600 MHz spectrometer. ^{1}H and ^{13}C chemical shifts are reported relative to the residual solvent peak and are given in ppm (δ). Data are reported as follows: chemical shift (multiplicity (singlet (s), broad singlet (br s), doublet (d), doublet of doublets (dd), doublet of doublet of doublets (ddd), triplet (t), doublet of triplets (dt), quartet (q), doublet of quartets (dq), pentet (p), heptet (h), multiplet (m)), coupling constants [Hz], integration, assignment). HR-ESI-MS spectra were recorded on a Bruker Daltronics microTOF II in positive or negative mode.

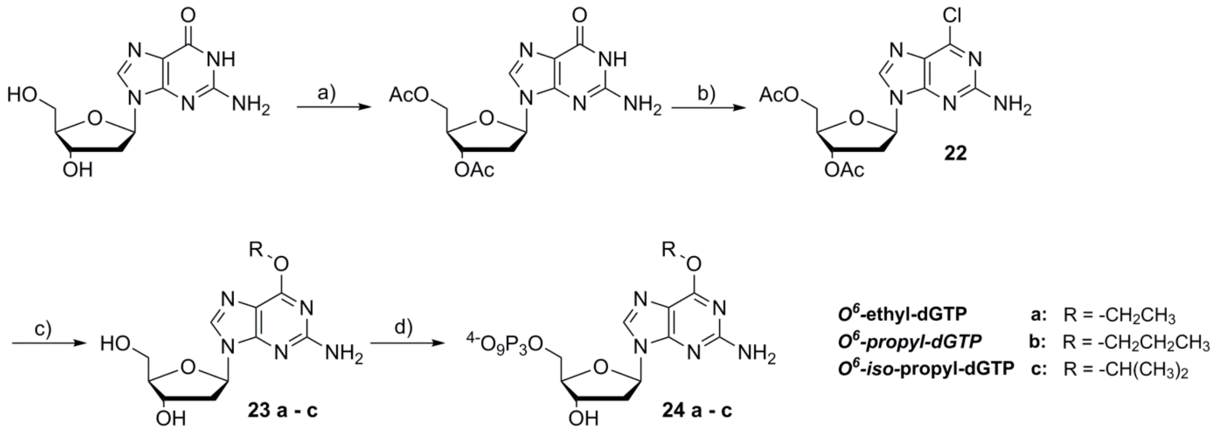

Figure S2: Synthesis scheme of modified nucleotides **24 a - c**. Reagents and conditions: a) Ac_2O, DMAP, Et_3N, MeCN, RT, 30 min, quant. b) $POCl_3$, tetraethylammonium chloride, *N,N*-dimethylaniline, MeCN, reflux, 15 min, 63 % c) 1 M NaOR in R-OH, reflux, 2 - 16 h, R = -CH_2CH_3 83 %, R = -$CH_2CH_2CH_3$ 78 %, R = -$CH(CH_3)_2$ 91 % d) *N,N,N´,N´*-Tetramethyl-1,8-naphthalenediamine, $POCl_3$, $(Bu_3NH)_2H_2P_2O_7$, *n*Bu_3N, trimethyl phosphate, 0.1 M TEAB, 0 °C, 30 min, *O*^{6}-ethyl-dGTP 22 %, *O*^{6}-propyl-dGTP 19 %, *O*^{6}-*iso*-propyl-dGTP 35 %

3´, 5´-Di-*O*-acetyl-2´-deoxyguanosine[2]

2´-Deoxyguanosine (5 g, 19.0 mmol, 1.0 eq.), DMAP (0.229 g, 1.9 mmol, 0.1 eq.) and triethylamine (7.3 ml, 57.0 mmol, 3.0 eq.) were dissolved in acetonitrile (100 ml). Acetic anhydride (5.39 ml, 57.0 mmol, 3.0 eq.) was added and the reaction mixture was stirred for 3 h at room temperature. The reaction was stopped by addition of 1 ml methanol and the solution was concentrated to dryness. The remaining white solid was co-evaporated using acetonitrile and washed with a mixture of ethanol and diethyl ether (1/1) to yield a white solid in quantitative yield (6.7 g, 19.0 mmol). ^{1}H-NMR (400 MHz, DMSO-d_6): δ = 10.65 (br s, 1H, -NH-), 7.91 (s, 1H, H-8), 6.48 (br s, 2H, -NH_2), 6.13 (dd, *J* = 5.9 Hz, 8.6 Hz, 1H, H-1´), 5.29 (dt, *J* = 2.0 Hz, 6.1 Hz, 1H, H-3´), 4.29 – 4.24 (m, 1H, H-5´a), 4.21 – 4.16 (m, 2H, H-4´, H-5´b), 2.91 (ddd, *J* = 6.3 Hz, 8.7 Hz, 14.6 Hz, 1H, H-2´a), 2.45 (ddd, *J* = 2.0 Hz, 5.9 Hz, 14.0 Hz, 1H, H-2´b), 2.08 (s, 3H, -CH_3), 2.03 (s, 3H, -CH_3) ppm. ^{13}C-NMR (100 MHz, DMSO-d_6): 170.6, 170.5, 157.1, 154.2, 151.6, 135.6, 117.3, 83.1, 81.9, 75.0, 64.1, 35.9, 21.3, 21.0 ppm. HR-ESI-MS $[M+1H]^+$: m/z calculated: 352.1252, m/z found: 352.1235.

3´, 5´-Di-*O*-acetyl-6-deoxy-6-chloro-2´-deoxyguanosine (22)[2]

To a suspension of 3´,5´-di-*O*-acetyl-2´-deoxyguanosine (4.0 g, 11.4 mmol, 1.0 eq.) in acetonitrile (60.0 ml) were added tetraethylammonium chloride (2.83 g, 17.0 mmol, 1.5 eq.) and *N,N*-dimethylaniline (8.7 ml, 68.3 mmol, 6.0 eq.). The reaction mixture was cooled to 0 °C and phosphoryl chloride (6.2 ml, 68.3 mmol, 6.0 eq.) was added dropwise. The reaction mixture was stirred at room temperature for 10 min. The mixture was heated to reflux for 15 min in a preheated oil bath, afterwards immediately cooled with an ice bath and quickly concentrated to dryness. The remaining phosphoryl chloride was slowly hydrolysed by addition of 30 ml of ice water under cooling. The solution was stirred for 20 min and extracted with ethyl acetate. The combined organic layers were washed with a saturated aqueous solution of sodium hydrogencarbonate and dried over $MgSO_4$, concentrated *in vacuo* and the crude residue was purified by column flash chromatography using methylene chloride with 2 % methanol yielding a white foam in 63 % yield (2.66 g, 7.2 mmol). ^{1}H-NMR (400 MHz, $CDCl_3$): 7.91 (s, 1H, H-8), 6.28 (dd, *J* = 6.2 Hz, 7.6 Hz, 1H, H-1´), 5.43 (dt, *J* = 2.3 Hz, 6.0 Hz, 1H, H-3´), 5.16 (br s, 2H, -NH_2), 4.46 (dd, *J* = 6.1 Hz, 13.4 Hz, 1H, H-5´a), 4.39 – 4.34 (m, 2H, H-4´, H-5´b), 2.97 (ddd, *J* = 6.4 Hz, 7.8 Hz, 14.2 Hz, 1H, H-2´a), 2.56 (ddd, *J* = 2.6 Hz, 6.1 Hz, 14.2 Hz, 1H, H-2´b), 2.14 (s, 3H, -C(O)-C**H_3**), 2.08 (s, 3H, -C(O)-C**H_3**) ppm. ^{13}C-NMR (100 MHz, $CDCl_3$): 170.6, 170.2, 159.0, 153.1, 151.8, 140.4, 126.0, 84.8, 82.5, 74.5, 63.7, 36.9, 20.9, 20.8 ppm. HR-ESI-MS $[M+1H]^+$: m/z calculated: 370.091, m/z found: 370.090.

Alkylation at position 6: General procedure A:

2´,3´-Di-*O*-acetyl-6-deoxy-6-chloro-2´-deoxyguanosine (**23**) (1.0 eq.) was dissolved in freshly prepared 1 M solution of the respective sodium alkoxide (30.0 eq.) in the corresponding alcohol and stirred under reflux overnight. After cooling to room temperature, the reaction mixture was concentrated to dryness, dissolved in water and the pH was adjusted to pH 7 using acetic acid. The aqueous solution was extracted with ethyl acetate and the organic layer was concentrated *in vacuo*. The remaining crude residue was further purified by column

flash chromatography using methylene chloride with 4 % methanol. The products were obtained as colourless foams.

2´-Deoxy-*O*⁶-ethyl-guanosine (23 a):

Yield: 83 % (265 mg, 0.9 mmol). ^{1}H-NMR (400 MHz, DMSO-d_6): 8.07 (s, 1H, H-8), 6.38 (br s, 2H, -NH_2), 6.21 (dd, *J* = 6.1 Hz, 7.8 Hz, 1H, H-1´), 5.25 (d, *J* = 4.0 Hz, 1H, 3´-OH), 4.98 (t, *J* = 5.7 Hz, 1H, 5´-OH), 4.45 (q, *J* = 7.1 Hz, 2H, -O^6-C**H$_2$**-CH_3), 4.35 (dq, *J* = 3.1 Hz, 6.1 Hz, 1H, H-3´), 3.82 (dt, *J* = 2.9 Hz, 4.4 Hz, 1H, H-4´), 3.57 (dt, *J* = 5.1 Hz, 11.7 Hz, 1H, H-5´a), 3.50 (ddd, *J* = 4.4 Hz, 5.8 Hz, 11.7 Hz, 1H, H-5´b), 2.58 (ddd, *J* = 3.1 Hz, 5.8 Hz, 7.8 Hz, 1H, H-2´a), 2.20 (ddd, *J* = 3.0 Hz, 6.0 Hz, 13.1 Hz, 1H, H-2´b), 1.35 (t, *J* = 7.1 Hz, 3H, -O^6-CH_2-C**H$_3$**) ppm. ^{13}C-NMR (100 MHz, DMSO-d_6): 160.8, 160.2, 154.3, 138.1, 114.4, 88.1, 83.2, 71.2, 62.2, 62.0, 39.5, 15.0 ppm. HR-ESI-MS $[M+1H]^+$: m/z calculated: 296.1353 $[M+1H]^+$, m/z found: 296.1342 $[M+1H]^+$

2´-Deoxy-*O*⁶-propyl-guanosine (23 b):

Yield: 78 % (260 mg, 0.8 mmol). ^{1}H-NMR (600 MHz, DMSO-d_6): 8.07 (s, 1H, H-8), 6.39 (br s, 2H, -NH_2), 6.21 (dd, *J* = 6.2 Hz, 7.6 Hz, 1H, H-1´), 5.27 (d, *J* = 3.8 Hz, 1H, 3´-OH), 4.99 (t, *J* = 5.4 Hz, 1H, 5´-OH), 4.36 – 4.34 (m, 3H, H-3´, -O^6-CH_2-), 3.82 (dt, *J* = 2.6 Hz, 4.4 Hz, 1H, H-4´), 3.58 – 3.55 (m, 1H, H-5´a), 3.52 – 3.48 (m, 1H, H-5´b), 2.58 (ddd, *J* = 5.7 Hz, 7.9 Hz, 13.4 Hz, 1H, H-2´a), 2.20 (ddd, *J* = 2.9 Hz, 5.9 Hz, 13.1 Hz, 1H, H-2´b), 1.77 (q, *J* = 7.1 Hz, 2H, -C**H$_2$**-CH_3), 0.97 (t, *J* = 7.4 Hz, 3H, -CH_3) ppm. ^{13}C-NMR (150 MHz, DMSO-d_6): 160.9, 160.2, 154.3, 138.0, 114.4, 88.0, 83.2, 71.2, 67.6, 62.2, 40.0, 22.3, 10.8 ppm. HR-ESI-MS $[M+1H]^+$: m/z calculated: 310.1524, m/z found: 310.1500.

2´-Deoxy-*O*⁶-*iso*-propyl-guanosine (23 c):

Yield: 91 % (304 mg, 1.0 mmol). ^{1}H-NMR (400 MHz, DMSO-d_6): 8.05 (s, 1H, H-8), 6.34 (br s, 2H, -NH_2), 6.20 (dd, *J* = 6.5 Hz, 7.9 Hz, 1H, H-1´), 5.48 (h, *J* = 6.1 Hz, 1H, -O^6-CH-), 5.25 (d, *J* = 4.0 Hz, 1H, 3´-OH), 4.98 (t, *J* = 5.7 Hz, 1H, 5´-OH), 4.35 (dq, *J* = 2.9 Hz, 6.0 Hz, 1H, H-3´), 3.82 (dt, *J* = 2.7 Hz, 4.5 Hz, 1H, H-4´), 3.57 (dt, *J* = 5.0 Hz, 11.7 Hz, 1H, H-5´a), 3.50 (ddd, *J* = 4.4 Hz, 5.8 Hz, 11.7 Hz, 1H, H-5´b), 2.57 (ddd, *J* = 5.7 Hz, 7.9 Hz, 13.3 Hz, 1H, H-2´a), 2.20 (ddd, *J* = 2.9 Hz, 5.9 Hz, 13.1 Hz, 1H, H-2´b), 1.34 (d, *J* = 2.5 Hz, 3H, -CH_3), 1.33 (d, *J* = 2.5 Hz, 3H, -CH_3) ppm. ^{13}C-NMR (100 MHz, DMSO-d_6): 160.4, 160.2, 154.3, 138.0, 114.6, 88.1, 83.3, 71.3, 68.7, 62.2, 40.0, 22.4, 22.4 ppm. HR-ESI-MS $[M+1H]^+$: m/z calculated: 310.1510, m/z found: 310.1500.

Triphosphorylation: General procedure B:

Typical reaction scales range from 40 mg to 120 mg of starting nucleoside. The respective nucleoside (1.0 eq.) and proton sponge (*N,N,N´,N´*-tetramethyl-1,8-naphthalenediamine) (1.5 eq.) were dried *in vacuo,* dissolved in dry trimethyl phosphate (1 ml per 20 mg of starting nucleoside) at room temperature and cooled to 0 °C. Phosphorous oxychloride (1.2 eq.) was

added dropwise at 0 °C and the mixture was stirred under nitrogen atmosphere. After 30 min TLC showed complete conversion of starting material and 0.5 M solution of $(Bu_3NH)_2H_2P_2O_7$ in anhydrous DMF (5.0 eq.) and $n$$Bu_3N$ (10.0 eq.) were added simultaneously to the mixture. The mixture was warmed to room temperature and stirred for 30 min. Then 0.1 M aqueous triethyl ammonium bicarbonate buffer (pH 7.5) (10 ml) was added and the mixture was stirred for further 30 min. The aqueous layer was washed with ethyl acetate several times to remove trimethyl phosphate and then concentrated to dryness. The residue was dissolved in water and purified by ion exchange chromatography (DEAE Sephadex A25, buffer A: 0.1 M TEAB, buffer B: 1 M TEAB, linear gradient: 0 % B to 100 % B) and further purified using reversed phase (RP)-HPLC (Nucleodur RP C18-HTec, buffer A: 50 mM TEAA, buffer B: acetonitrile, linear gradient: 5 % B to 100 % B). The triphosphates were concentrated to dryness. To remove the triethylammonium acetate the residues were dissolved in water and freeze dried several times.

2´-Deoxy-*O*^{6}-ethyl-guanosine-5´-*O*-triphosphate (*O*^{6}-ethyl-dGTP) (24 a):

Yield: 22 % (48.2 µmol). ^{1}H-NMR (400 MHz, D_2O): 8.24 (s, 1H, H-8), 6.39 (dd, J = 6.5 Hz, 7.4 Hz, 1H, H-1´), 4.83 – 4.80 (m, 1H, H-3´), 4.55 (q, J = 7.1 Hz, 2H, -*O*^{6}-C**H₂**-CH$_3$), 4.30 – 4.27 (m, 1H, H-4´), 4.26 – 4.21 (m, 1H, H-5´a), 4.17 (ddd, J = 4.0 Hz, 5.4 Hz, 11.4 Hz, 1H, H-5´b), 2.85 (ddd, J = 6.5 Hz, 7.4 Hz, 14.0 Hz, 1H, H-2´a), 2.55 (ddd, J = 3.4 Hz, 6.3 Hz, 14.0 Hz, 1H, H-2´b), 1.46 (t, J = 7.1 Hz, 3H, -*O*^{6}-CH$_2$-C**H$_3$**) ppm. ^{13}C-NMR (100 MHz, D_2O): 160.7, 160.0, 152.1, 138.3, 112.7, 85.8, 83.9, 71.0, 65.4, 64.0, 38.8, 13.6 ppm. ^{31}P-NMR (162 MHz, D_2O): -10.00 (d, J = 19.4 Hz, 1P, α-P), -11.34 (d, J = 19.1 Hz, 1P, γ-P), -23.1 (t, J = 19.9 Hz, β-P) ppm. HR-ESI-MS $[M\text{-}1H]^-$: m/z calculated: 534.0198, m/z found: 534.0181 $[M\text{-}1H]^-$. ε = 8900 L mol^{-1}cm^{-1} (λ = 281 nm)[3]

2´-Deoxy-*O*^{6}-propyl-guanosine-5´-*O*-triphosphate (*O*^{6}-propyl-dGTP) (24 b):

Yield: 19 % (50.1 µmol). ^{1}H-NMR (400 MHz, D_2O): 8.23 (s, 1H, H-8), 6.35 (t, J = 6.9 Hz, 1H, H-1´), 4.78 – 4.76 (m, 1H, H-3´), 4.40 (t, J = 6.6 Hz, 2H, -*O*^{6}-CH$_2$-), 4.28 – 4.24 (m, 1H, H-4´), 4.23 – 4.13 (m, 2H, H-5´a/b), 2.83 (ddd, J = 6.3 Hz, 8.0 Hz, 13.8 Hz, 1H, H-2´a), 2.55 (ddd, J = 3.3 Hz, 6.2 Hz, 14.0 Hz, 1H, H-2´b), 1.83 (q, J = 7.1 Hz, 2H, -C**H$_2$**-CH$_3$), 1.03 (t, J = 7.4 Hz, 3H, -CH$_3$) ppm. ^{13}C-NMR (100 MHz, D_2O): 161.2, 160.0, 152.9, 138.5, 113.8, 85.6, 83.4, 71.2, 69.4, 65.5, 38.6, 21.7, 9.65 ppm. ^{31}P-NMR (162 MHz, D_2O): -11.00 (d, J = 20.0 Hz, 1P, α-P), -11.45 (d, J = 20.3 Hz, 1P, γ-P), -23.34 (t, J = 20.1 Hz, β-P) ppm. HR-ESI-MS $[M\text{-}1H]^-$: m/z calculated: 548.0354, m/z found: 548.0352 $[M\text{-}1H]^-$. ε = 9400 L mol^{-1}cm^{-1} (λ = 247 nm)[3]

2´-Deoxy-*O*^{6}-*iso*-propyl-guanosine-5´-*O*-triphosphate (*O*^{6}-*iso*-propyl-dGTP) (24 c):

Yield: 35 % (89.7 µmol). ^{1}H-NMR (400 MHz, D_2O): 8.23 (s, 1H, H-8), 6.35 (t, J = 6.9 Hz, 1H, H-1´), 5.43 (h, J = 6.2 Hz, 1H, -*O*^{6}-CH-), 4.78 – 4.76 (m, 1H, H-3´), 4.28 – 4.24 (m, 1H, H-4´), 4.21 – 4.12 (m, 2H, H-5´a/b), 2.81 (ddd, J = 6.1 Hz, 7.6 Hz, 13.8 Hz, 1H, H-2´a), 2.54 (ddd, J = 3.3 Hz, 6.3 Hz, 14.0 Hz, 1H, H-2´b), 1.40 (d, J = 6.6 Hz, 3H, -CH$_3$), 1.39 (d, J = 6.6 Hz, 3H, -CH$_3$) ppm. ^{13}C-NMR (100 MHz, D_2O): 160.6, 160.0, 152.9, 138.5, 114.0, 85.6, 83.4, 71.5,

71.1, 65.5, 38.6, 21.1 ppm. ^{31}P-NMR (162 MHz, D_2O): -11.00 (d, *J* = 20.0 Hz, 1P, α-P), -11.47 (d, *J* = 19.2 Hz, 1P, γ-P), -23.4 (t, *J* = 19.9 Hz, β-P) ppm. HR-ESI-MS $[M-1H]^-$: m/z calculated: 548.0354, m/z found: 548.0351 $[M-1H]^-$. ε = 9400 L $mol^{-1}cm^{-1}$ (λ = 247 nm)[3]

Synthesis of 3´-terminally modified primers:

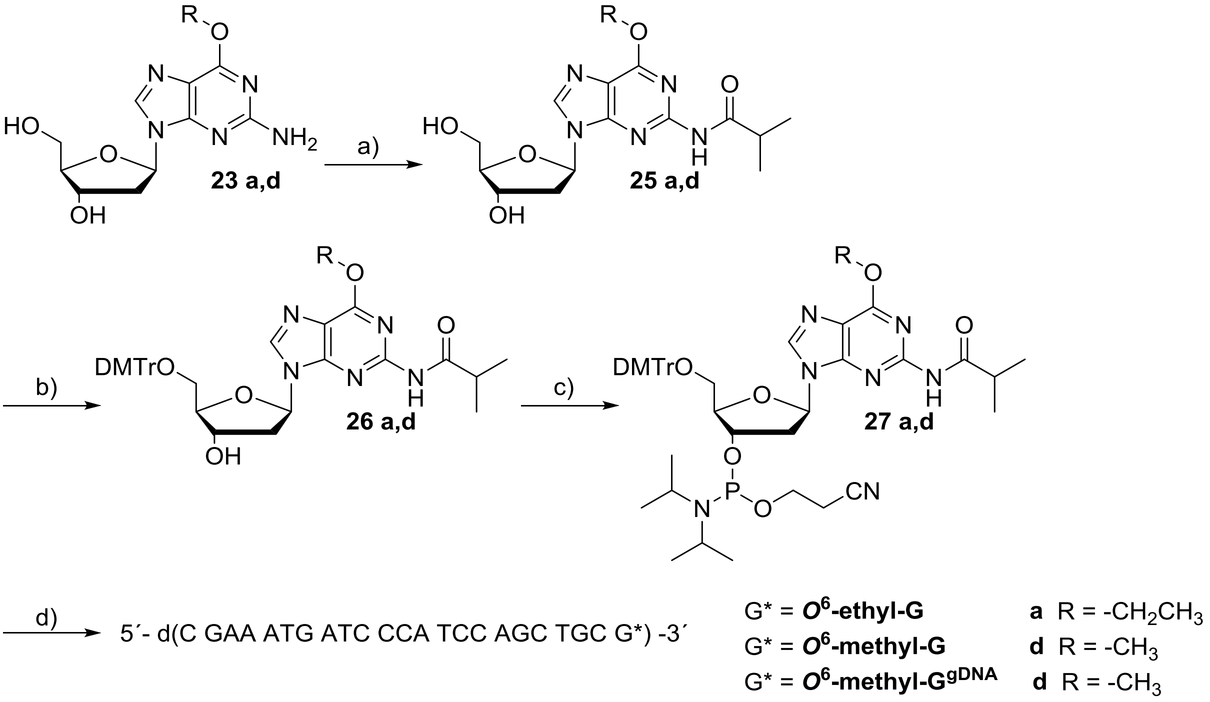

Figure S3: Synthesis scheme of the 3´-terminally modified ***O*^{6}-methyl-G** and ***O*^{6}-ethyl-G** primer. Reagents and conditions: a) Trimethylchlorosilane, isobutyric anhydride, pyridine, RT, overnight; water, RT, 15 min; 29 % aqueous ammonia solution, RT, 15 min, R = -CH_3 69 %, R = -CH_2CH_3 75 % b) 4,4´-Dimethoxytrityl chloride, pyridine, RT, overnight, R = -CH_3 63 %, R = -CH_2CH_3 69 % c) 2-Cyanoethyl diisopropylphosphoramidochloridite, $^{i}Pr_2EtN$, DCM, RT, 30 min, R = -CH_3 67 %, R = -CH_2CH_3 85 % d) Standard phosphoramidite DNA solid phase synthesis: 500 Å UniversalQ SynBase CPG, 0.1 M standard phosphoramidites; 33 % aq. NH_3, 55 °C, overnight; 80 % AcOH, RT, 2 h, *O*^{6}-methyl-G 9 %, *O*^{6}-ethyl-G 32 %, *O*^{6}-methyl-G^{gDNA}.

Iso-butyryl protection of *N*^{2}: General procedure C:[4]

The respective 2´-deoxy-*O*^{6}-alkyl-guanosine (2.0 mmol, 1 eq.) dried three times by co-evaporation with pyridine was suspended in dry pyridine (8 ml). Trimethylchlorosilane (10.0 mmol, 5 eq.) was added and the mixture was stirred for 4 h at room temperature. After TLC (CH_2Cl_2/ MeOH 10/1) showed complete conversion of starting material isobutyric anhydride (10.0 mmol, 5 eq.) was added and the mixture was stirred overnight at room temperature. Following, the reaction was cooled to 0 °C and water (3 ml) was added. After 15 min 29 % aqueous ammonia solution (3 ml) was added and the reaction was stirred for further 15 min. The reaction was concentrated *in vacuo* and the residue was dissolved in 100 ml of water. The mixture was washed with a mixture of ethyl acetate and diethylether (1/1). The aqueous layer was concentrated *in vacuo* and the residue was stirred in saturated sodium bicarbonate. The solution was concentrated to dryness and the residue was further purified by column flash chromatography using methylene chloride with up to 4 % methanol.

2´-Deoxy-*N*[2]-*iso*-butyryl-*O*[6]-methyl-guanosine (25 d):

Yield: 69 % (1.4 mmol). ^{1}H-NMR (400 MHz, DMSO-*d*$_6$): 10.34 (br s, 1H, -NH-), 8.43 (s, 1H, H-8), 6.33 (t, *J* = 6.9 Hz, 1H, H-1´), 5.30 (d, *J* = 3.3 Hz, 1H, 3´-OH), 4.88 (t, *J* = 5.4 Hz, 1H, 5´-OH), 4.42 (dq, *J* = 3.1 Hz, 6.3 Hz, 1H, H-3´), 4.08 (s, 3H, -O^6-CH_3), 3.87 – 3.82 (m, 1H, H-4´), 3.59 (dt, *J* = 5.4 Hz, 11.1 Hz, 1H, H-5´a), 3.54 -3.48 (m, 1H, H-5´b), 2.89 (h, *J* = 6.9 Hz, 1H, -C(O)-CH-), 2.70 (dt, *J* = 6.6 Hz, 13.1 Hz, 1H, H-2´a), 2.33 – 2.25 (m, 1H, H-2´b), 1.09 (d, *J* = 6.7 Hz, 6H, 2 x -CH_3) ppm. ^{13}C-NMR (100 MHz, DMSO-*d*$_6$): 175.5, 160.7, 152.9, 152.6, 141.4, 117.9, 88.3, 83.6, 71.2, 62.1, 54.3, 39.9, 34.8, 19.8, 19.8 ppm. HR-ESI-MS $[M+1H]^+$: m/z calculated: 352.1615, m/z found: 352.1613.

2´-Deoxy-*N*[2]-*iso*-butyryl-*O*[6]-ethyl-guanosine (25 a):

Yield: 75 % (1.5 mmol). ^{1}H-NMR (400 MHz, DMSO-*d*$_6$): 10.31 (br s, 1H, -NH-), 8.42 (s, 1H, H-8), 6.33 (t, *J* = 6.7 Hz, 1H, H-1´), 5.31 (d, *J* = 3.9 Hz, 1H, 3´-OH), 4.90 (t, *J* = 5.4 Hz, 1H, 5´-OH), 4.57 (q, 7.1 Hz, 2H, -O^6-C**H$_2$**-CH_3), 4.42 (dq, *J* = 3.3 Hz, 6.2 Hz, 1H, H-3´), 3.85 (dt, *J* = 2.8 Hz, 4.7 Hz, 1H, H-4´), 3.59 (dt, *J* = 5.0 Hz, 11.9 Hz, 1H, H-5´a), 3.52 (dt, *J* = 4.8 Hz, 11.6 Hz, 1H, H-5´b), 2.88 (h, *J* = 6.8 Hz, 1H, -C(O)-CH-), 2.69 (ddd, *J* = 5.7 Hz, 7.6 Hz, 13.2 Hz, 1H, H-2´a), 2.28 (ddd, *J* = 3.2 Hz, 6.2 Hz, 13.2 Hz, 1H, H-2´b), 1.40 (t, *J* = 7.0 Hz, 3H, -O^6-CH_2-C**H$_3$**), 1.09 (d, *J* = 6.8 Hz, 6H, 2 x -CH_3) ppm. ^{13}C-NMR (100 MHz, DMSO-*d*$_6$): 175.5, 160.4, 153.0, 152.5, 141.3, 117.9, 88.3, 83.6, 71.1, 63.1, 62.1, 39.9, 34.8, 19.8, 19.7, 14.9 ppm. HR-ESI-MS $[M+1H]^+$: m/z calculated: 366.1772, m/z found: 366.1761.

DMTr protection of 5´-OH: General procedure D:[5]

To a solution of dried, *N*[2]-*iso*-butyryl-protected nucleosides (1.0 mmol, 1.0 eq.) in pyridine (7 ml) was added 4,4`-dimethoxytrityl chloride (1.5 mmol, 1.5 eq.) and the mixture was stirred overnight at room temperature. After completion of the reaction methanol was added (10 ml) and stirred for 15 min. The solution was concentrated to dryness and purified using column flash chromatography with methylene chloride, 1 % triethylamine and 2 % methanol.

2´-Deoxy-5´-*O*-(4,4´,-dimethoxytrityl)-*N*[2]-*iso*-butyryl-*O*[6]-methyl-guanosine (26 d):

Yield: 63 % (0.6 mmol). ^{1}H-NMR (400 MHz, DMSO-*d*$_6$): 10.28 (br s, 1H, -NH-), 8.31 (s, 1H, H-8), 7.30 – 7.28 (m, 2H, $H_{arom,\ DMTr}$), 7.20 – 7.14 (m, 7H, $H_{arom,\ DMTr}$), 6.77 – 6.70 (m, 4H, $H_{arom,\ DMTr}$), 6.37 (t, *J* = 6.4 Hz, 1H, H-1´), 5.33 – 5.28 (m, 1H, 3´-OH), 4.53 (dq, *J* = 4.2 Hz, 6.4 Hz, 1H, H-3´), 4.08 (s, 3H, -O^6-CH_3), 3.99 – 3.93 (m, 1H, H-4´), 3.71 (s, 3H, -OCH_3), 3.70 (s, 3H, -OCH_3), 3.41 – 3.26 (m, 2H, H-5´a/b), 3.11 (dd, *J* = 3.3 Hz, 10.3 Hz, 1H, -C(O)-C**H**-$(CH_3)_2$), 2.95 – 2.80 (m, 1H, H-2´a), 2.34 (ddd, *J* = 4.7 Hz, 6.8 Hz, 13.4 Hz, 1H, H-2´b), 1.07 (t, *J* = 6.5 Hz, 6H, 2 x -CH_3) ppm. ^{13}C-NMR (100 MHz, DMSO-*d*$_6$): 175.3, 160.8, 158.4, 158.3, 152.8, 152.6, 145.4, 141.7, 136.1, 136.0, 130.2, 130.0, 127.0, 118.1, 113.4, 113.3, 86.4, 85.8, 83.8, 71.0, 64.9, 55.4, 55.4, 54.4, 40.0, 34.8, 19.8, 19.8 ppm. HR-ESI-MS $[M+1H]^+$: m/z calculated: 668.3079, m/z found: 668.3079.p

2´-Deoxy-5´-*O*-(4,4´,-dimethoxytrityl)-*N*^{2}-*iso*-butyryl-*O*^{6}-ethyl-guanosine (26 a):

Yield: 69 % (0.7 mmol). ^{1}H-NMR (400 MHz, DMSO-d_6): 10.24 (br s, 1H, -NH-), 8.30 (s, 1H, H-8), 7.30 – 7.28 (m, 2H, $H_{arom, DMTr}$), 7.20 – 7.15 (m, 7H, $H_{arom, DMTr}$), 6.78 – 6.71 (m, 4H, $H_{arom, DMTr}$), 6.36 (t, *J* = 6.4 Hz, 1H, H-1´), 5.33 – 5.27 (m, 1H, 3´-OH), 4.57 (h, *J* = 7.0 Hz, 2H, -*O*^{6}-C**H₂**-CH$_3$), 4.52 (dq, *J* = 4.5 Hz, 6.3 Hz, 1H, H-3´), 3.96 (dt, *J* = 3.4 Hz, 7.2 Hz, 1H, H-4´), 3.71 (s, 3H, -OCH$_3$), 3.70 (s, 3H, -OCH$_3$), 3.29 – 3.26 (m, 1H, H-5´a), 3.10 (dd, *J* = 3.2 Hz, 10.4 Hz, 1H, -C(O)-C**H**-(CH$_3$)$_2$), 2.93 – 2.72 (m, 2H, H-5´b, H-2´a), 2.38 – 2.29 (m, 1H, H-2´b), 1.23 (t, *J* = 7.2 Hz, 3H, -*O*^{6}-CH$_2$-C**H$_3$**), 1.07 (t, *J* = 6.4 Hz, 6H, 2 x -CH$_3$) ppm. ^{13}C-NMR (100 MHz, DMSO-d_6): 175.3, 160.4, 158.4, 158.3, 152.9, 152.6, 145.4, 141.5, 136.1, 130.2, 130.0, 128.2, 128.1, 127.0, 118.1, 113.4, 113.3, 86.7, 85.8, 83.8, 71.0, 64.9, 63.0, 55.4, 55.3, 52.5, 46.2, 34.8, 19.8, 14.9, 12.2, 7.7 ppm. HR-ESI-MS [M+1H]$^{+}$: m/z calculated: 668.3079, m/z found: 668.3065.

Conversion to phosphoramidite: General procedure E:[5]

2-Cyanoethyl diisopropylphosphoramidochloridite (1.2 eq.) was added to a mixture of protected nucleoside (1.0 eq.), and $^{i}Pr_2EtN$ (4.0 eq.) in acid free dry methylene chloride at 0 °C. The solution was stirred for 30 min at room temperature under N_2. After TLC showed complete conversion of starting material, the solution was concentrated to dryness and purified by column flash chromatography using ethyl acetate with 1 % triethylamine.

2´-Deoxy-5´-*O*-(4,4´,-dimethoxytrityl)-*N*^{2}-*iso*-butyryl-*O*^{6}-methyl-guanosine-3´-(2-cyanoethyl-di-*iso*-propyl-phosphoramidite (26 d):

Yield: 67 % (0.1 mmol). ^{1}H-NMR (400 MHz, d_3-acetonitrile): isomeric mixture: 8.41 + 8.40 (br s, 1H, -NH-), 7.99 (s, 1H, H-8), 7.37 – 7.32 (m, 2H, $H_{arom, DMTr}$), 7.24 – 7.17 (m, 7H, $H_{arom, DMTr}$), 6.76 – 6.68 (m, 4H, $H_{arom, DMTr}$), 6.34 + 6.33 (t, *J* = 6.4 Hz, 1H, H-1´), 4.91 – 4.83 (m, H-3´), 4.23 – 4.14 (m, 1H, H-4´), 4.10 (s, 3H, -*O*^{6}-CH$_3$), 3.84 – 8.64 (m, 4H, 2 x -N-C**H**-(CH$_3$)$_2$, -C**H₂**-CH$_2$-CN), 3.75 – 3.72 (m, 6H, 2 x -OCH$_3$), 3.63 – 3.54 (m, 2H, H5´a/b), 3.44 – 3.38 (m, 1H, -C(O)-C**H**-(CH$_3$)$_2$), 3.30 – 3.23 (m, 1H, H2´a), 3.05 (ddd, *J* = 4.6 Hz, 6.4 Hz, 13.3 Hz, 1H, H2´b), 2.64 (t, *J* = 6.2 Hz, 1H, -CH$_2$-CN), 2.51 (t, *J* = 6.2 Hz, 1H, -CH$_2$-CN), 1.18 – 1.10 (m, 18H, 6 x -CH$_3$) ppm. ^{31}P-NMR (162 MHz, d_3-acetonitrile): 148.29 + 148.05 (s, 1P) ppm. HR-ESI-MS [M-1H+1Cl]$^{-}$: m/z calculated: 854.4001, m/z found: 854.3991.

2´-Deoxy-5´-*O*-(4,4´,-dimethoxytrityl)-*N*^{2}-*iso*-butyryl-*O*^{6}-ethyl-guanosine-3´-(2-cyanoethyl-di-*iso*-propyl-phosphoramidite (26 a):

Yield: 85 % (0.1 mmol). ^{1}H-NMR (400 MHz, d_6-acetone): isomeric mixture: 9.04 + 9.03 (br s, 1H, -NH-), 8.17 + 8.16 (s, 1H, H-8), 7.44 – 7.40 (m, 2H, $H_{arom, DMTr}$), 7.31 – 7.27 (m, 4H, $H_{arom, DMTr}$), 7.23 – 7.16 (m, 3H, $H_{arom, DMTr}$), 6.80 – 6.72 (m, 4H, $H_{arom, DMTr}$), 6.46 + 6.46 (t, *J* = 6.3 Hz, 1H, H-1´), 5.02 – 4.94 (m, 1H, H-4´), 4.58 (q, *J* = 7.1 Hz, -*O*^{6}-C**H₂**-CH$_3$), 4.28 + 4.23 (dt, *J* = 3.8 Hz, 6.9 Hz, 1H, H-3´), 3.95 – 3.81 (m, 1H, H-5´a), 3.77 – 3.76 (m, 6H, 2 x -OCH$_3$), 3.70 – 3.62 (m, 2H, 2 x -N-C**H**-(CH$_3$)$_2$), 3.56 + 3.53 (t, *J* = 6.1 Hz, 2H, -CH$_2$-CN), 3.38 + 3.34 (dd, *J* = 3.6 Hz, 10.4 Hz, 1H, H-5´b), 3.20 – 3.13 (m, 1H, H-2´a), 2.68 (ddd, *J* = 4.1 Hz, 6.7 Hz, 13.9

Hz, 1H, H-2´b), 2.63 + 2.62 (t, J = 6.1 Hz, 2H, -C**H₂**-CH_2-CN), 1.44 (t, J = 7.1 Hz, 3H, -O^6-CH_2-C**H₃**), 1.21 – 1.16 (m, 18 H, 6 x -CH_3) ppm. ^{31}P-NMR (162 MHz, d_6-acetone): 148.1 + 147.3 (s, 1P) ppm. HR-ESI-MS $[M\text{-}1H\text{+}1Cl]^-$: m/z calculated: 902.3778, m/z found: 902.3617.

Oligonucleotide synthesis

200 nmol scale coupling and oxidation was performed using a DNA synthesizer (Model 392, Applied Biosystems) using standard parameters (DMT-on mode) for solid phase oligonucleotide synthesis. Standard phosphoramidites were diluted in abs. acetonitrile to a final concentration of 0.10 M and the synthesis was performed on 500 Å UniversalQ SynBase CPG (Link Technologies) with loading densities of approximately 41 µmol/g. Using extended coupling times of 5 min for couplings with modified phosphoramidites, average coupling yields were always higher than 95 %. The synthesized oligonucleotides were cleaved from the solid support and deprotected using 33 % aq. NH_3 overnight at 55 °C. The DMT-containing oligonucleotides were purified by RP-HPLC (RP-18; solvent: 0.05 M TEAA with a gradient of acetonitrile from 5 – 50 % over 20 min. flow rate: 2 ml/min). The oligonucleotide was concentrated and the mixture was treated with 80 % AcOH for 2 h at room temperature. The detritylated oligonucleotide was again purified by RP-HPLC (RP-18; solvent: 0.05 M TEAA with a gradient of acetonitrile from 5 – 70 % over 20 min. flow rate: 2 ml/min), concentrated and lyophylized using a Speed Vac evaporator. The resulting colourless solids were stored at -20 °C.

Table S1: Mass and yields of synthesized oligonucleotides bearing modified nucleotides at the 3´-terminus.

**G***	mass calc. [M-1H]$^-$	mass found [M-1H]$^-$	yield
O^6-methyl-G	7006.2125	7006.2227	9 %
O^6-ethyl-G	7021.2491	7021.3000	32 %
Sequence: 5´-d(CGAAATGATCCCATCCAGCTGC**G***)-3´			
O^6-methyl-G^{gDNA}	6224.0219	6224.0792	5 %
Sequence: 5´-d(CTTGGTGAGACTGGTAGAC**G***)-3´			

Biochemical methods

Oligonucleotides were purchased from Biomers in HPLC grade and used directly for primer extension experiments.

Quantification of oligonucleotides

Quantification of oligonucleotides was conducted by measuring absorbance at 260 nm in water.

5´-Radioactive labelling of oligonucleotides

DNA oligonucleotide primers were radioactively labelled at the 5´-terminus by usage of [γ-^{32}P]-ATP and T4 PNK. The reactions contained 0.4 µM primer, 1 x PNK reaction buffer, 0.8 µCi/µl [γ-^{32}P]-ATP and 0.4 U/µl T4 PNK in a total volume of 50 µl. The reaction mixture was incubated at 37 °C and stopped after 1 h by denaturation of the T4 PNK for 2 min at 95 °C. Buffer and excess [γ-^{32}P]-ATP were removed by gelfitration (MicroSpin Sephadex G-25). Addition of 20 µl of unlabelled primer (10 µM) led to a final concentration of 3 µM of diluted radioactive labelled primer.

Primer extension assay

The mixture of 150 nM of a [γ-^{32}P]-labeled primer (5´-d(CGA AAT GAT CCC ATC CAG CTG C)-3´, or 5´-d(CGA AAT GAT CCC ATC CAG CTG CN)-3´, N = G/G*) and 200 nM of either template (5´-d(CCG CTG CCC ACC AGC CAT CAT GTC GGA CCC CGC GGT CAA CGX GCA GCT GGA TGG GAT CAT TTC GGA CT)-3´, X = C/5mC) in buffer (*KOD exo*$^-$: 50 mM Tris-HCl pH 8.0, 16 mM $(NH_4)_2SO_4$, 2.5 mM $MgCl_2$, 0.1 % Tween 20; *KlenTaq*: 50 mM Tris-HCl, pH 9.2, 16 mM $(NH_4)_2SO_4$, 1.75 mM $MgCl_2$, 0.1 % Tween 20) was heated to 95 °C for 2 min and subsequently cooled to 4 °C for annealing. The respective DNA polymerase was added and the reaction was started by addition of 50 µM of the respective dNTP at 55 °C. Reactions (10 µl) were stopped after the desired incubation time by addition of 10 µl stop solution (80 % (v/v) formamide, 20 mM EDTA, 0.25 % (w/v) bromophenol blue, 0.25 % (w/v) xylene cyanol) and analysed by 12 % or 15 % denaturing PAGE. Visualization was performed by phosphorimaging.

Gel electrophoresis

Denaturing polyacrylamide gels (12 % or 15 %) were prepared by polymerization of a solution of bisacrylamide/acrylamide (12 % or 15 %) and urea (8.3 M) in TBE buffer using peroxodisulfate (APS, 0.08 %) and *N,N,N´,N´*-tetramethylethylene diamine (TEMED, 0.04 %). After initiation of polymerization the solution was filled in a sequencing gel chamber (Bio-Rad) and left for polymerization for at least 30 min. After addition of TBE buffer (1 x) to the electrophoresis unit, the gel was prewarmed by electrophoresis at 100 W for 20 min before samples were applied to the gel. After electrophoresis at 100 W for approximately 2.5 h, the gel was transferred to Whatman filter paper and dried at 80 °C under reduced pressure using a gel dryer (Model 583, Bio-Rad). The dried gel was exposed to an imager screen overnight and read out was performed using a molecular imager (FX, Bio-Rad). Quantification was done using the Bio-Rad software Image Lab.ink.

PAGE analysis of single nucleotide incorporation PEx of dGTP and *O*[6]-alkyl-dGTP derivatives opposite C or 5mC employing *KlenTaq*:

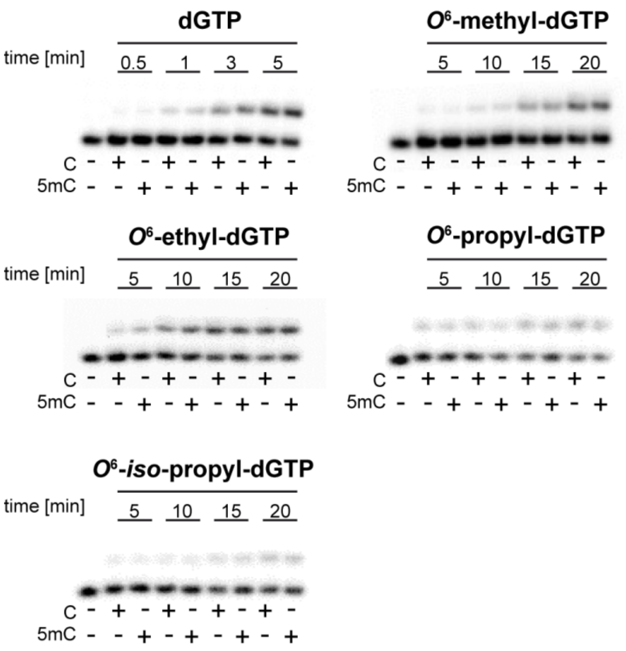

Figure S4: PAGE analysis of single-nucleotide incorporation primer extension experiments of nucleotides dGTP, *O*[6]-methyl-dGTP, *O*[6]-ethyl-dGTP, *O*[6]-propyl-dGTP and *O*[6]-*iso*-propyl-dGTP opposite a template containing C in comparison to a template containing 5mC employing *KlenTaq* DNA polymerase. 50 µM dGTP or dG*TP and 0.1 nM *KlenTaq* were used, reactions were stopped after indicated time points.

PAGE analysis of single nucleotide incorporation PEx of dGTP and *O*^{6}-alkyl-dGTP derivatives opposite C, T, G and A employing *KOD exo*$^-$:

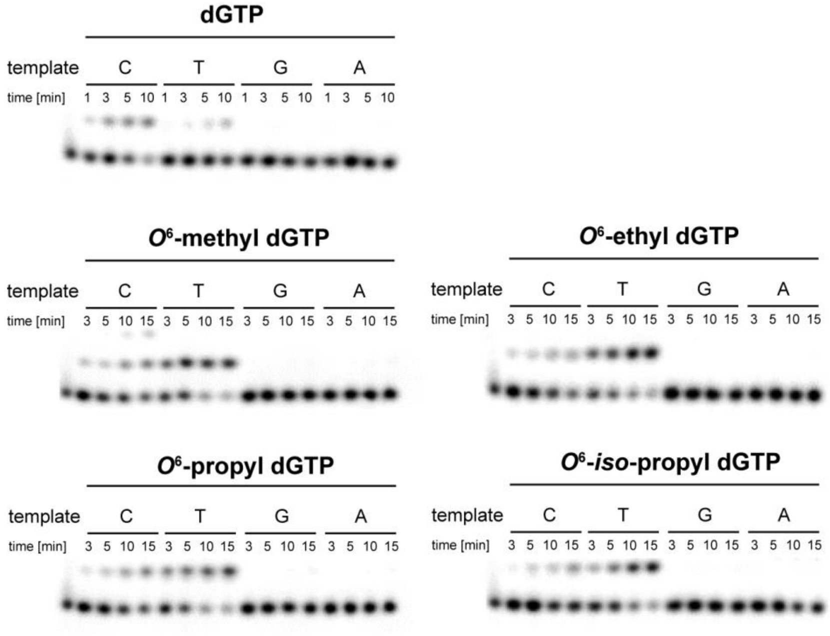

Figure S5: PAGE analysis of single-nucleotide incorporation primer extension experiments of nucleotides dGTP, *O*^{6}-methyl-dGTP, *O*^{6}-ethyl-dGTP, *O*^{6}-propyl-dGTP and *O*^{6}-*iso*-propyl-dGTP opposite a template containing C, T, G or A employing *KOD exo*$^-$ DNA polymerase. 50 µM dGTP or dG*TP and 5 nM *KOD exo*$^-$ were used, reactions were stopped after indicated timepoints.

PAGE analysis of single-nucleotide incorporation PEx of primers bearing G, *O*^{6}-methyl-G or *O*^{6}-ethyl-G at the 3'-end opposite C or 5mC employing *KOD exo*$^-$:

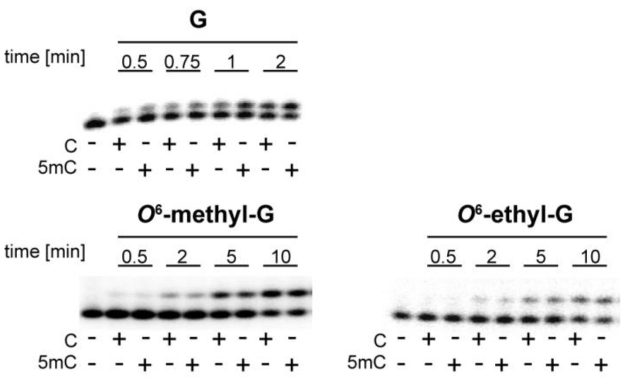

Figure S6: PAGE analysis of single-nucleotide incorporation primer extension experiments of primers bearing G, *O*^{6}-methyl-G and *O*^{6}-ethyl-G at the 3'-end opposite C or 5mC catalyzed by *KOD exo*$^-$. 50 µM dCTP and 10 nM *KOD exo*$^-$ were used, reactions were stopped after indicated timepoints.

Enzyme kinetics

Steady-state kinetics of *KOD exo*$^-$ and *KlenTaq* were measured under single completed hit conditions.[6] Single-nucleotide incorporation for the respective dNTP, primers in complex with either template (see above) was performed as described, analysed by 12 % or 15 % denaturating PAGE and visualized by phosphorimaging. Concentrations of the respective DNA polymerase were chosen, so that less than 20 % of the applied primer was extended. The rate of single-nucleotide incorporation was determined at various dNTP concentrations for different incubation times varying from 10 s – 330 s. The amount of extended primer was plotted against incubation time for each examined dNTP concentration. For kinetic analysis, the reaction velocities divided through DNA polymerase concentrations were plotted against the employed dNTP concentrations. Using OriginPro8, experimental data was fit to a hyperbolic equation [velocity] = v_{Max}[dNTP]/(K_M+[dNTP]) to determine K_M and k_{cat}.

Steady-state kinetic analysis of dGTP and *O*^{6}-alkyl-dGTP derivatives incorporation oppsite C or 5mC employing *KOD exo*$^-$:

Table S2: Steady-state kinetic analysis of single-nucleotide incorporation opposite C/5mC by *KOD exo*$^-$ DNA polymerase.

incorporated nucleotide	template	k_{cat} [a] [s^{-1}]	K_M [a] [µM]	k_{cat}/K_M [a] [s^{-1} μM^{-1}]
dGTP	C	5.9 ± 0.1	4.0 ± 0.3	1.5 ± 0.1
dGTP	5mC	3.5 ± 0.1	3.3 ± 0.3	1.1 ± 0.1
O^6-methyl-dGTP	C	3.338 ± 0.001	20.7 ± 2.6	0.16 ± 0.02
O^6-methyl-dGTP	5mC	1.12 ± 0.6	18.2 ± 1.6	0.062 ± 0.010
O^6-ethyl-dGTP	C	2.27 ± 0.06	15.8 ± 2.0	0.14 ± 0.02
O^6-ethyl-dGTP	5mC	0.91 ± 0.06	27.4 ± 4.0	0.033 ± 0.007
O^6-propyl-dGTP	C	2.55 ± 0.04	24.1 ± 3.1	0.105 ± 0.015
O^6-propyl-dGTP	5mC	1.47 ± 0.02	15.2 ± 1.4	0.097 ± 0.011
O^6-*iso*-propyl-dGTP	C	2.44 ± 0.14	78.8 ± 8.8	0.031 ± 0.005
O^6-*iso*-propyl-dGTP	5mC	1.19 ± 0.09	56.0 ± 7.7	0.021 ± 0.004

Sequence primer: 5´-d(CGAAATGATCCCATCCAGCTGC)-3´
[a] Data points derive from triplicates. ± describes SD.

a)

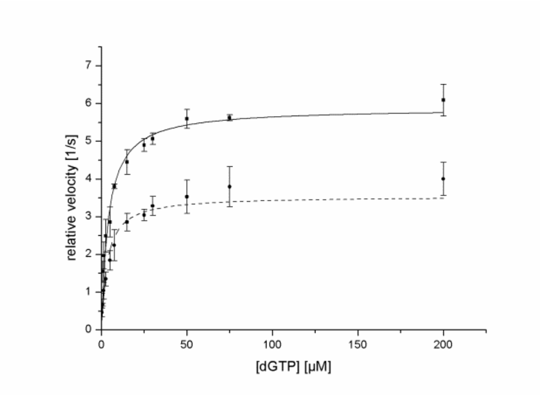

b)

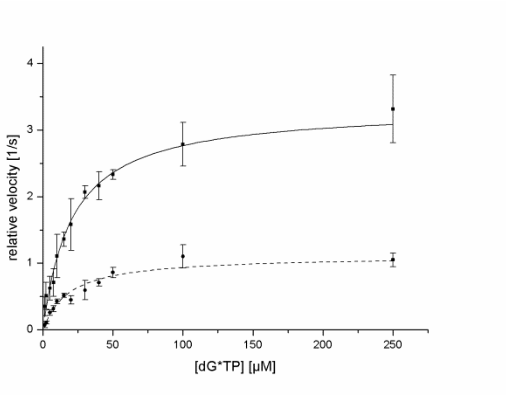

c)

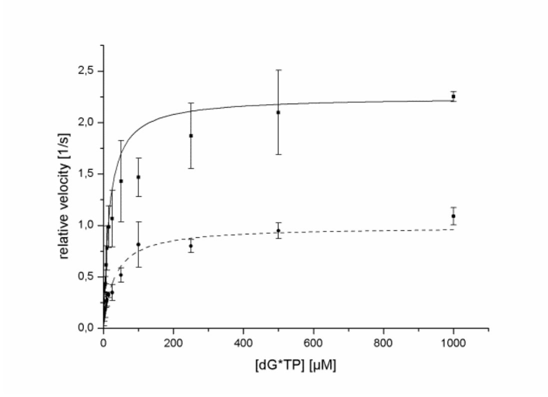

d)

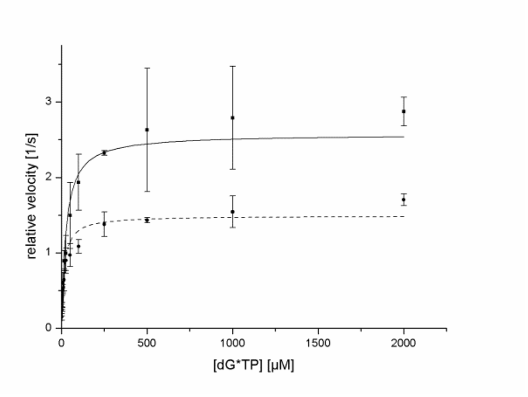

e)

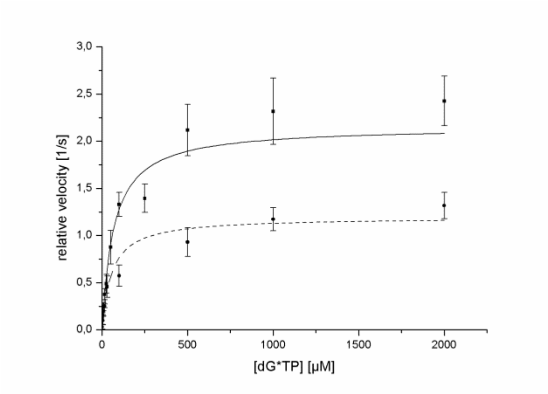

Figure S7: Steady-state kinetics of single-nucleotide incorporation of natural or modified dGTP (dGTP/dG*TP) opposite C (solid line) or 5mC (dashed line). Incorporation using a) natural dGTP, b) O^6-methyl-dGTP, c) O^6-ethyl-dGTP, d) O^6-propyl-dGTP and e) O^6-*iso*-propyl-dGTP as substrates.

Steady-state kinetic analysis of dCTP incorporation in extension of primers bearing G, *O*^{6}-methyl-G or *O*^{6}-ethyl-G at the 3´-terminus paired with C or 5mC employing *KlenTaq*:

Table S3: Steady-state kinetic analysis of single-nucleotide incorporation (dCTP) using the primers bearing G, *O*^{6}-methyl-G and *O*^{6}-ethyl-G at the 3´-terminus paired with C/5mC and *KlenTaq* DNA polymerase.

3´ terminally G/G* primer	template	k_{cat} [a] [s^{-1}]	K_M [a] [µM]	k_{cat}/K_M [a] [s^{-1} $µM^{-1}$]
G	C	514.4 ± 0.3	58.9 ± 5.8	8.7 ± 1.3
G	5mC	689.2 ± 0.2	22.7 ± 1.9	30.3 ± 3.4
O^6-methyl-G	C	1.32 ± 0.07	306.21 ± 42.47	0.0043 ± 0.0008
O^6-methyl-G	5mC	5.71 ± 0.55	197.92 ± 35.30	0.0289 ± 0.0079
O^6-ethyl-G	C	1.73 ± 0.24	261.22 ± 42.71	0.0066 ± 0.0002
O^6-ethyl-G	5mC	7.10 ± 0.91	305.47 ± 66.49	0.0233 ± 0.0080

Sequence primer: 5´-d(CGAAATGATCCCATCCAGCTGC**G/G***)-3´
[a] Data points derive from triplicates. ± describes SD.

a)

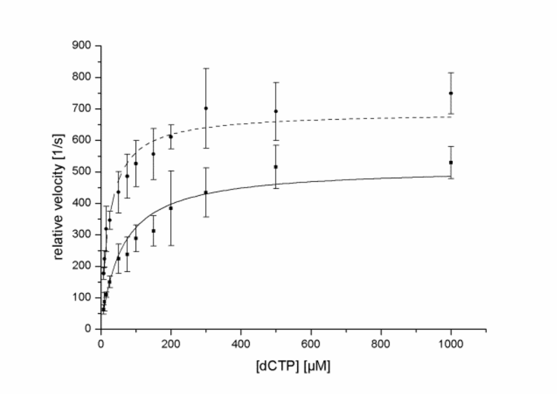

b)

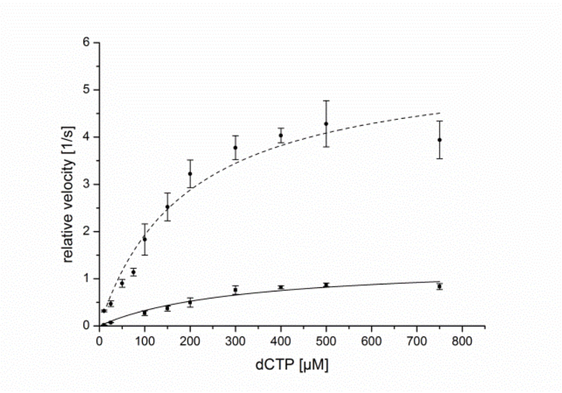

c)

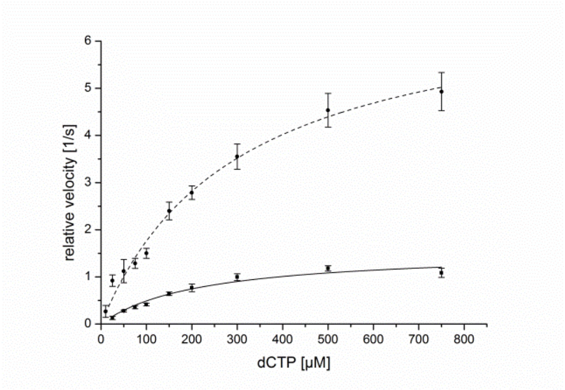

Figure S8: Steady-state kinetics of single-nucleotide incorporation (dCTP) using the primers bearing G, *O*^{6}-methyl-G or *O*^{6}-ethyl-G at the 3´-terminus paired with C (solid line) or 5mC (dashed line). Extension of the primer bearing a) G, b) *O*^{6}-methyl-G and c) *O*^{6}-ethyl-G at the 3´-terminus.

PCR experiments

The reaction mixture contained 200 µM dNTPs (each), 100 nM forward primer (5´-d(CTT GGT GAG ACT GGT AGA CN)-3´, N = G/G*), 100 nM reverse primer (5´-d(CAA CCA GCT CAG TCC AGC AGA ACG)-3´), 100 nM *KlenTaq* DNA polymerase, 100 nM aptamer, 1 x sybr green I (Sigma), 500 mM betaine and 5 ng/µl either human HeLa gDNA or CpG methylated HeLa gDNA in 1x reaction buffer (50 mM Tris-HCl pH 9.2, 16 mM $(NH_4)_2SO_4$, 2.5 mM $MgCl_2$, 0.1 % Tween 20). The data were obtained from respectively 10 µl reaction mixtures using the Biorad CFX384™ Real-Time system (C-1000 Touch Thermal Cycler). Initial denaturation was performed at 95 °C for 2 min followed by amplification over 50 cycles with denaturation at 95 °C for 15 s. The annealing and elongation steps were performed at 58.3 °C for 30 s and 72 °C for 1 min.

NMR Spectra:

2´-Deoxy-O^6-ethyl-guanosine (23 a):

^{1}H-NMR (400 MHz, DMSO-d_6):

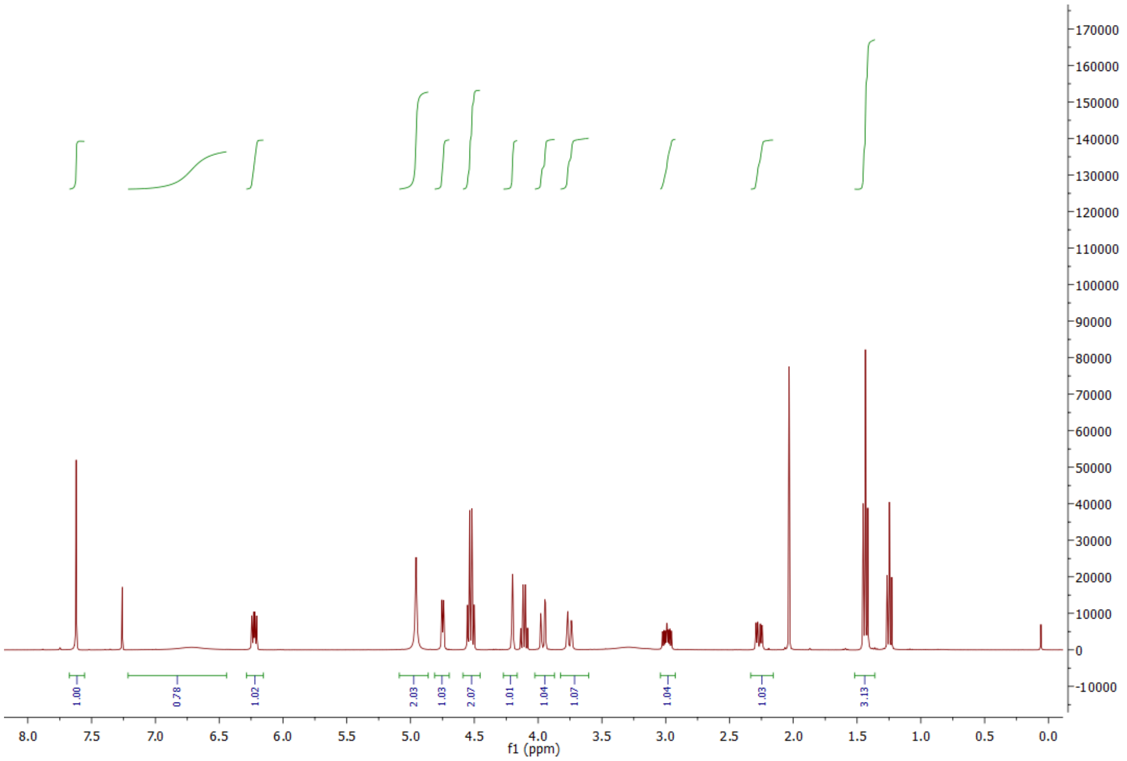

^{13}C-NMR (100 MHz, DMSO-d_6):

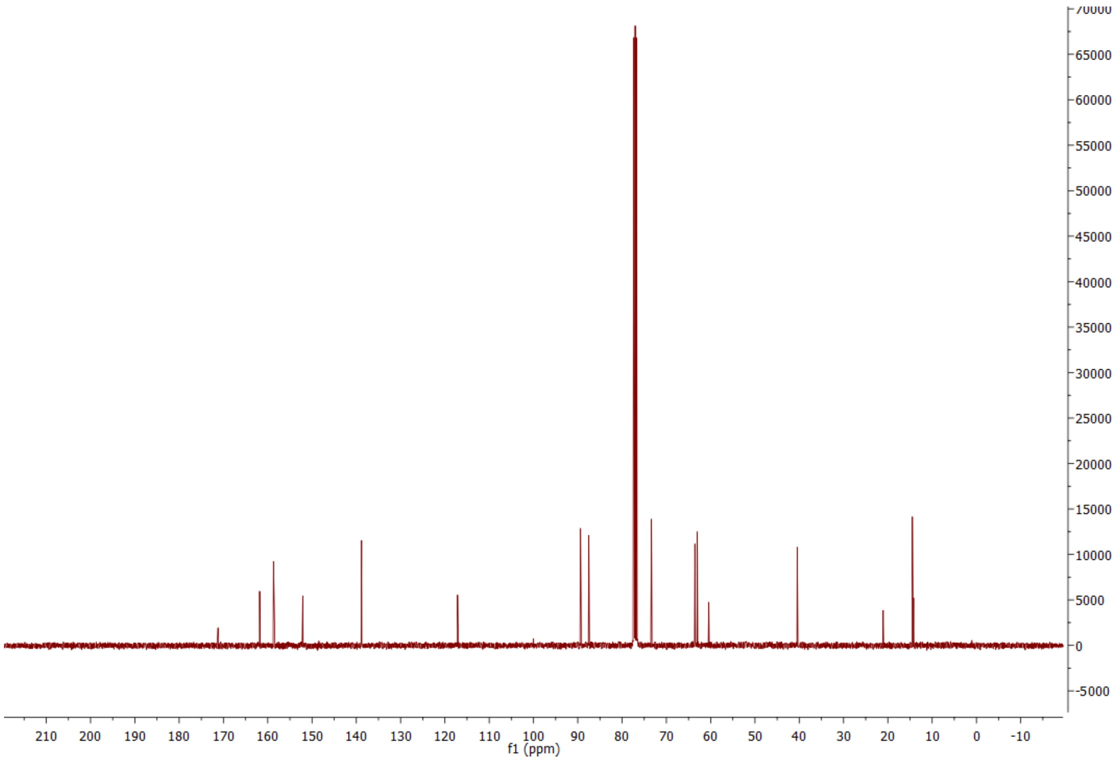

2´-Deoxy-*O*^{6}-propyl-guanosine (23 b):

^{1}H-NMR (400 MHz, DMSO-d_6):

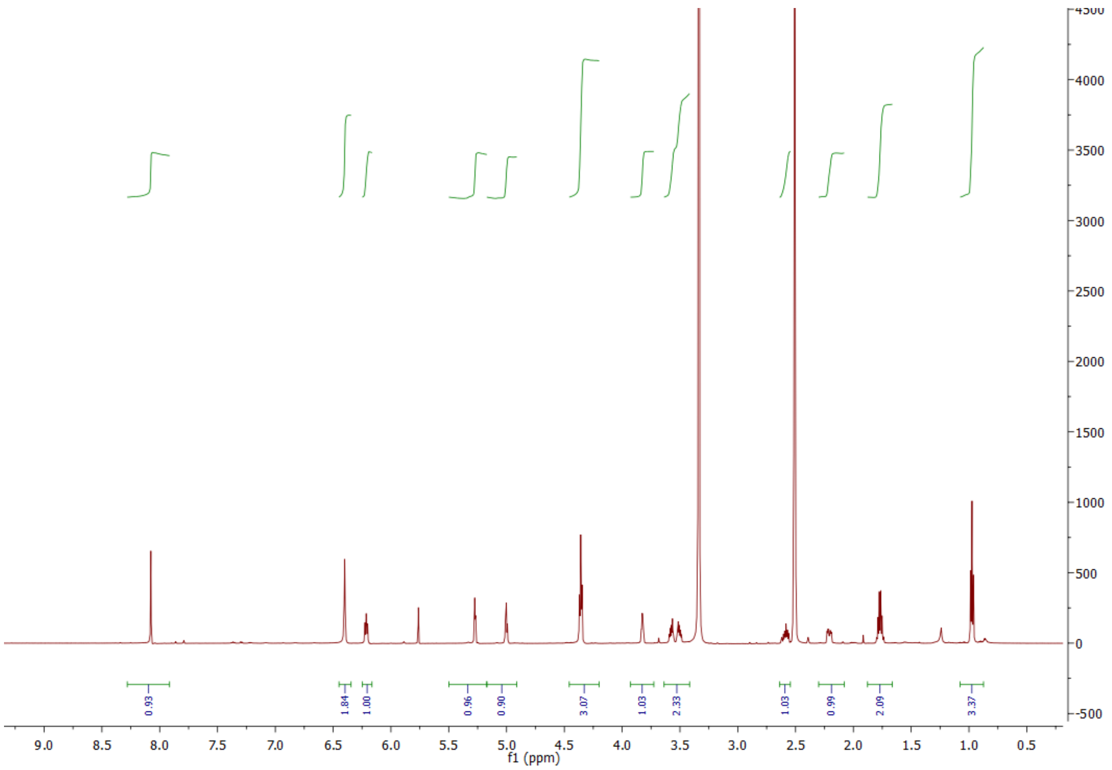

^{13}C-NMR (100 MHz, DMSO-d_6):

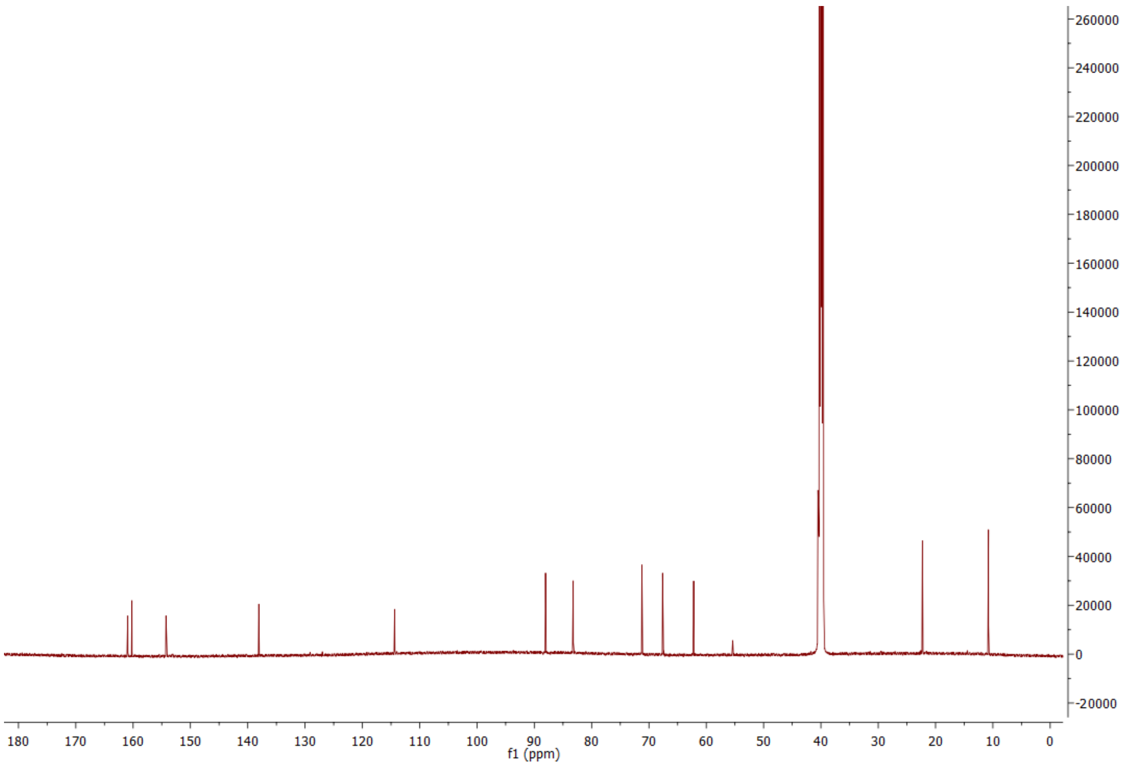

2´-Deoxy-*O*^{6}-*iso*-propyl-guanosine (23 c):

^{1}H-NMR (400 MHz, DMSO-d_6):

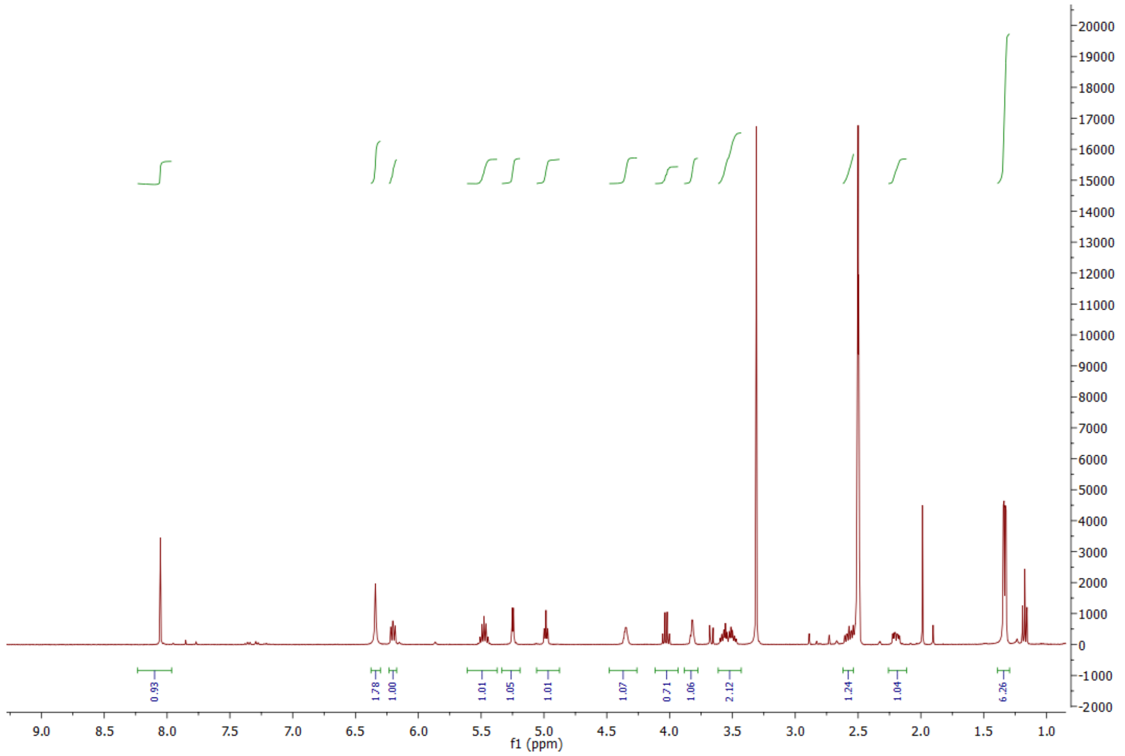

^{13}C-NMR (100 MHz, DMSO-d_6):

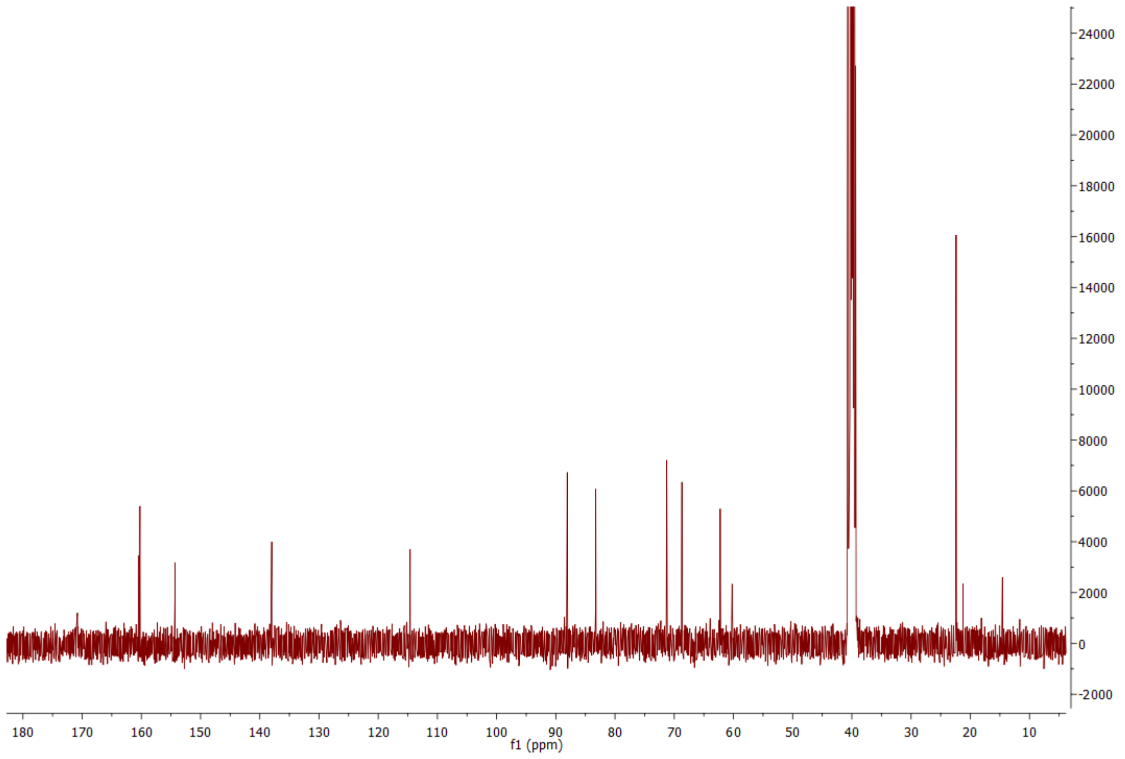

2´-Deoxy-*O*⁶-ethyl-guanosine-5´-*O*-triphosphate (*O*⁶-ethyl-dGTP) (24 a):

^{1}H-NMR (400 MHz, D_2O):

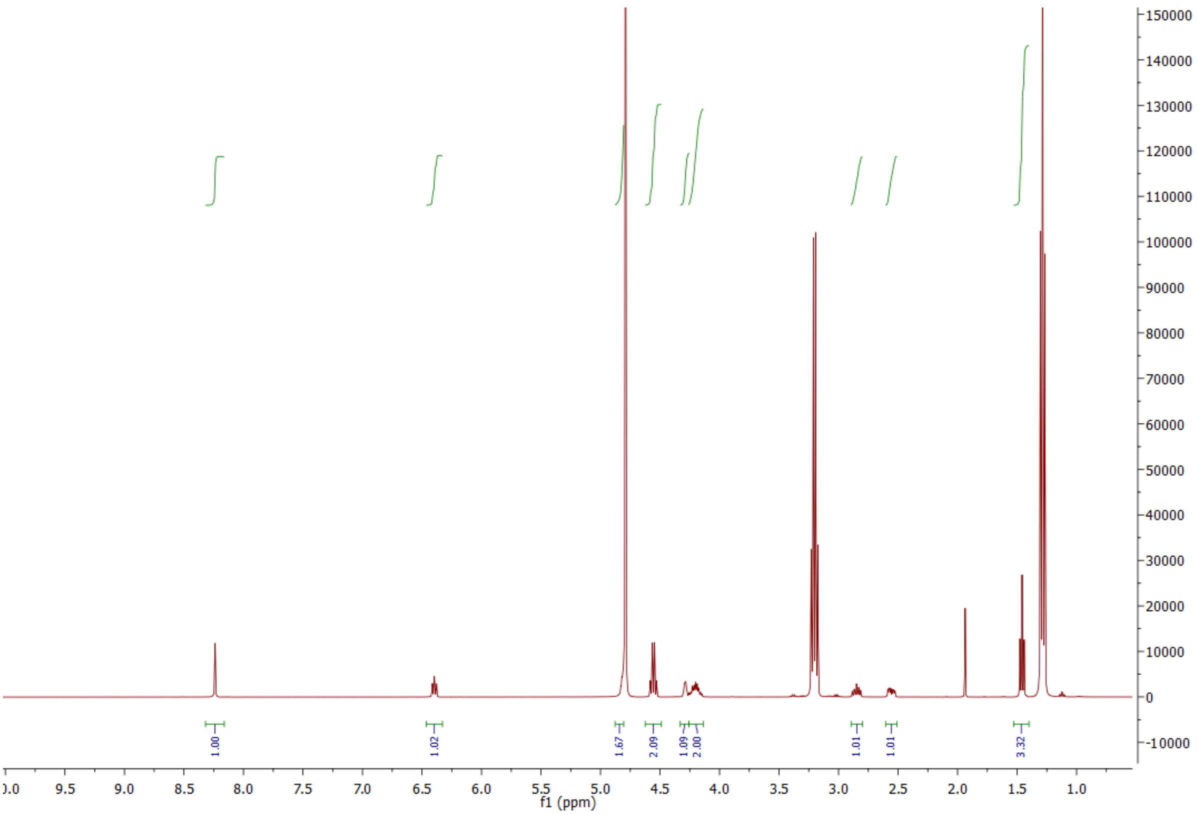

^{31}P-NMR (162 MHz, D_2O):

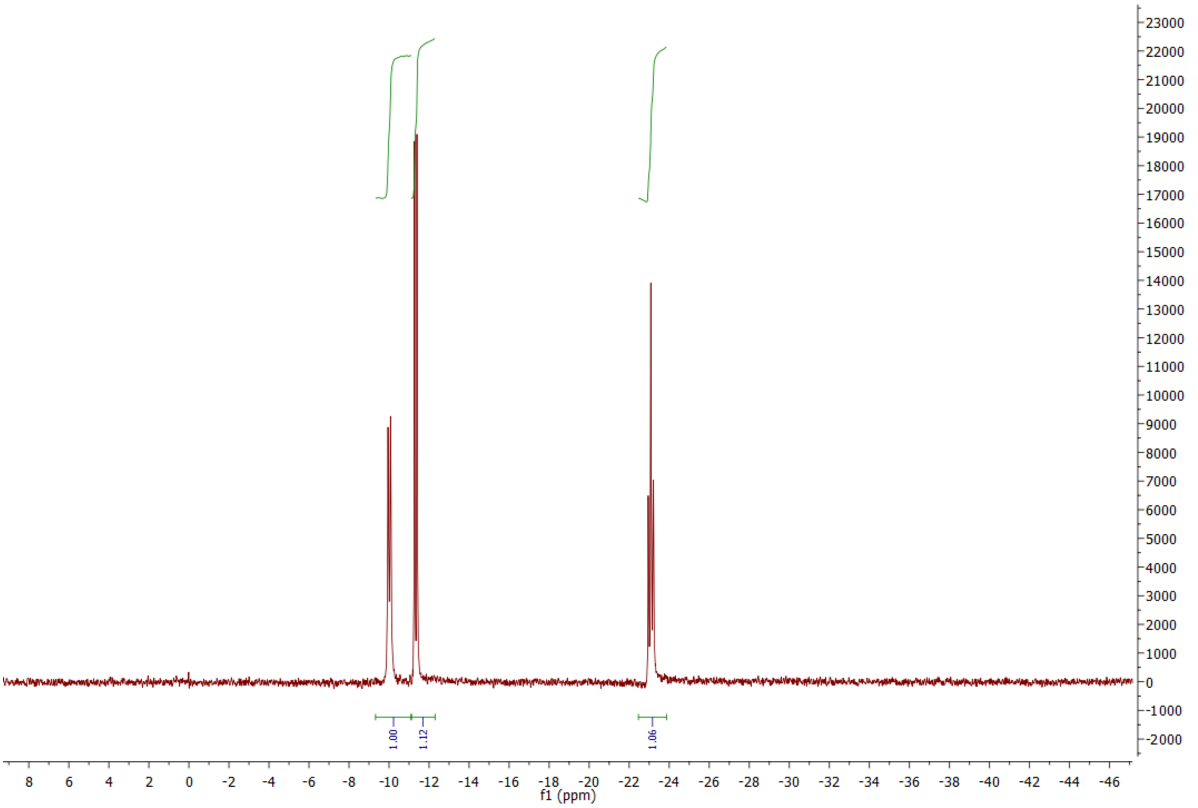

^{13}C-NMR (100 MHz, D_2O):

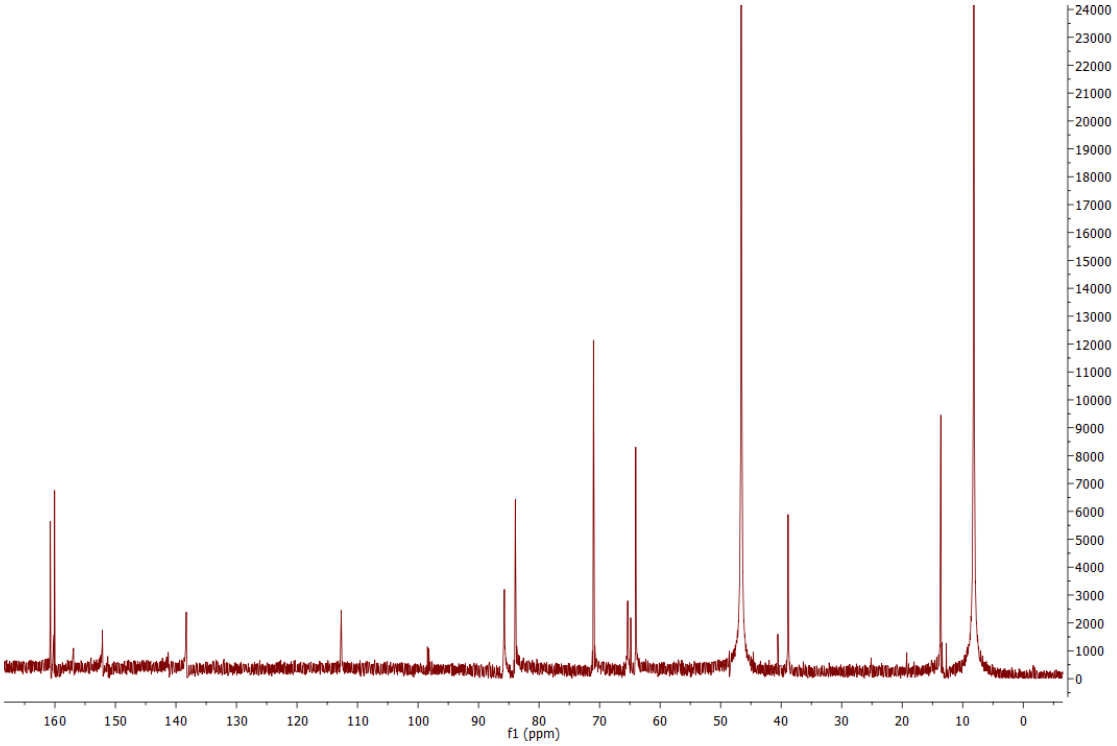

2´-Deoxy-*O*⁶-propyl-guanosine-5´-*O*-triphosphate (*O*⁶-propyl-dGTP) (24 b):

^{1}H-NMR (400 MHz, D_2O):

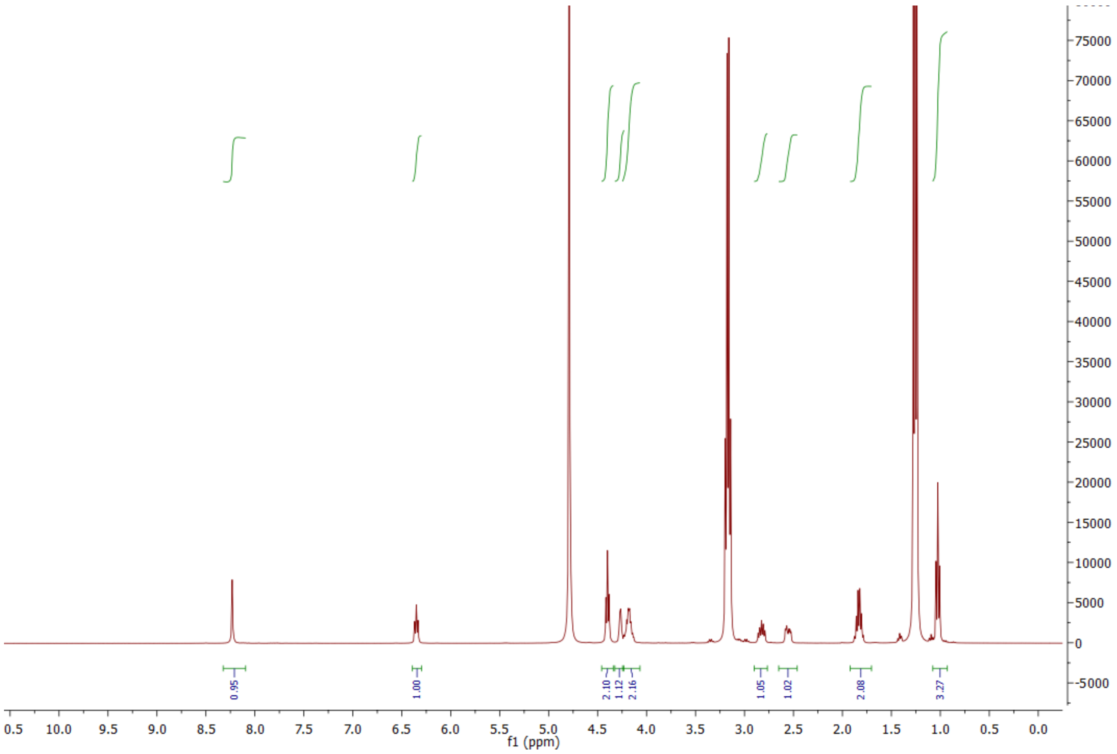

^{31}P-NMR (162 MHz, D_2O):

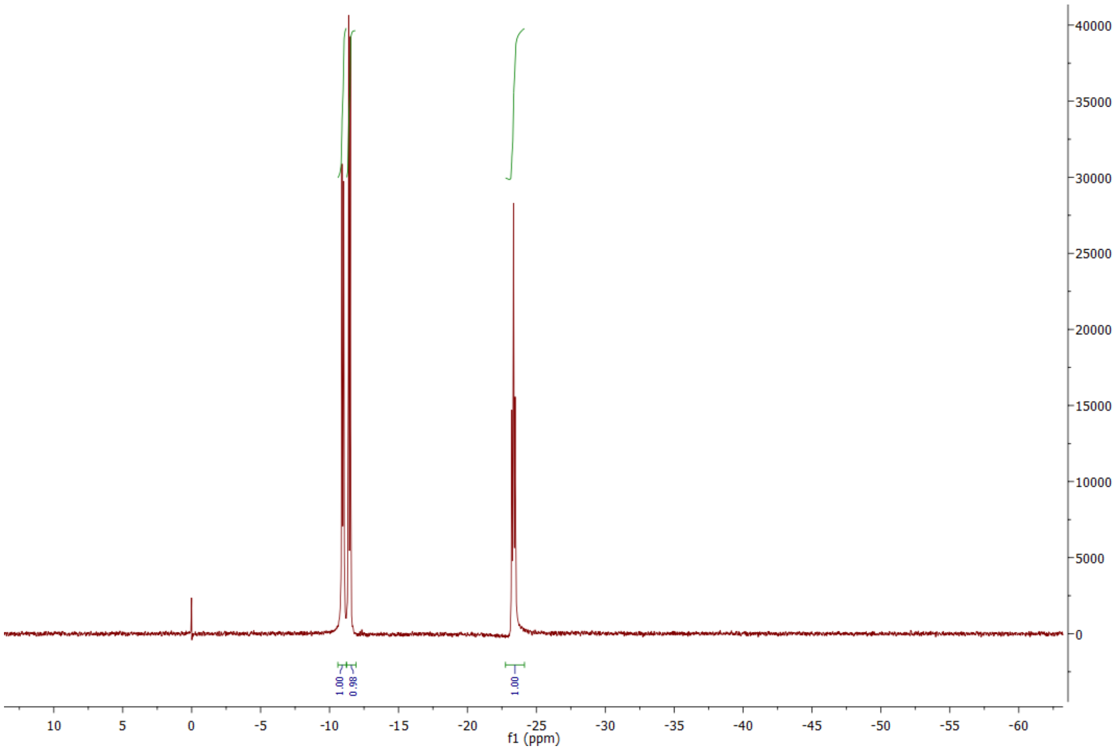

^{13}C-NMR (100 MHz, D_2O):

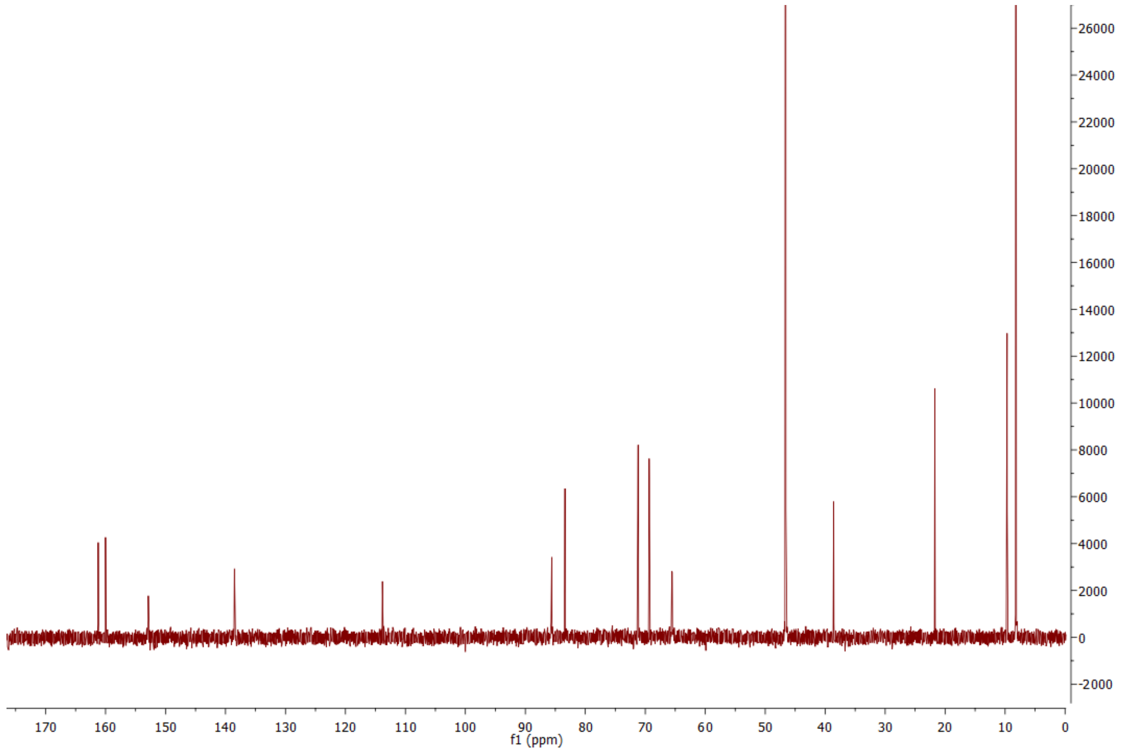

2´-Deoxy-*O*^{6}-*iso*-propyl-guanosine-5´-*O*-triphosphate (*O*^{6}-*iso*-propyl-dGTP) (24 c):

^{1}H-NMR (400 MHz, D_2O):

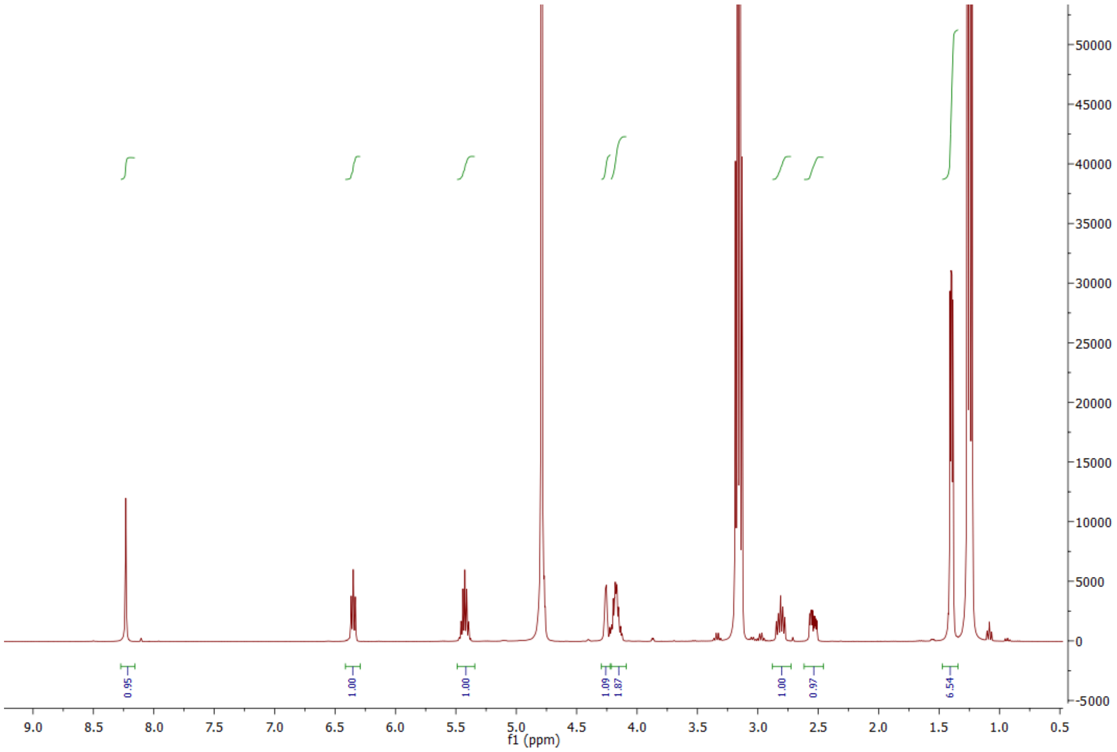

^{31}P-NMR (162 MHz, D_2O):

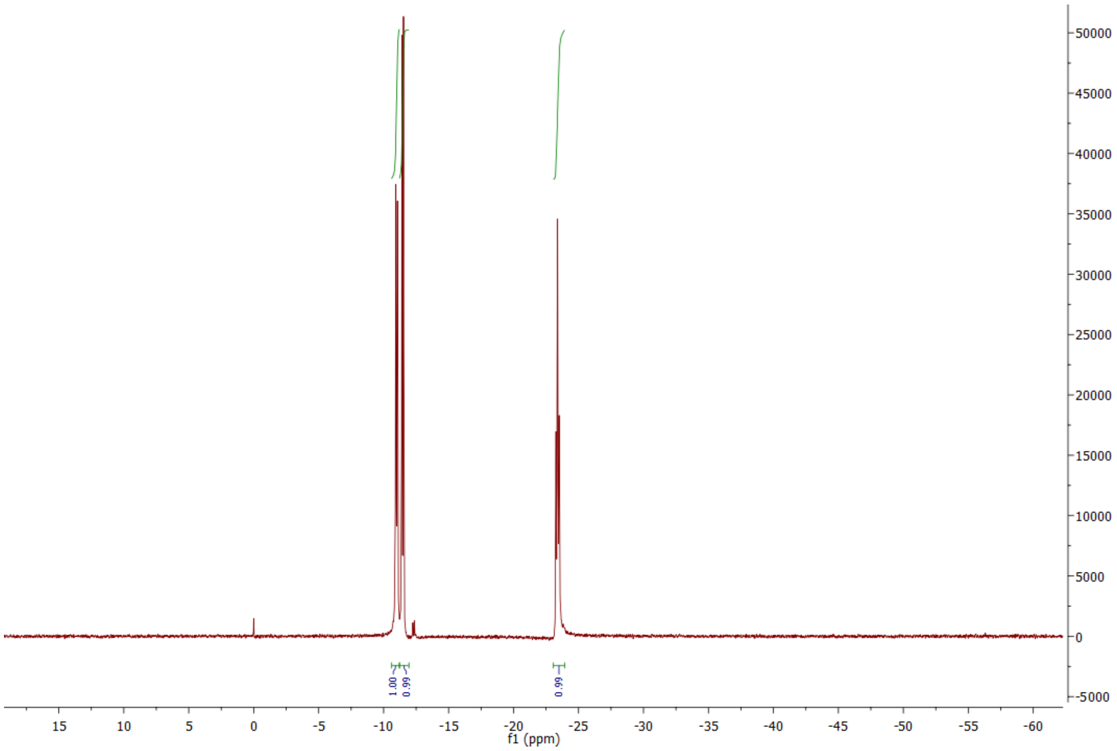

^{13}C-NMR (100 MHz, D_2O):

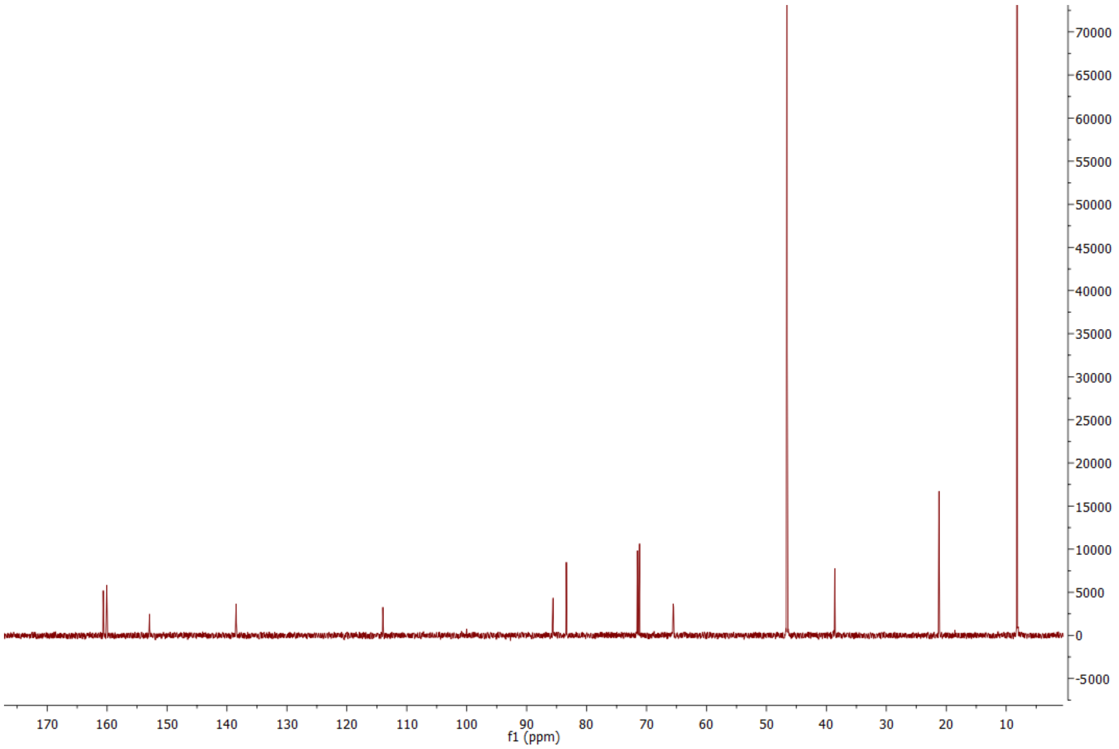

HR-MS Spectra of modified primers:

O[6]-methyl-G:

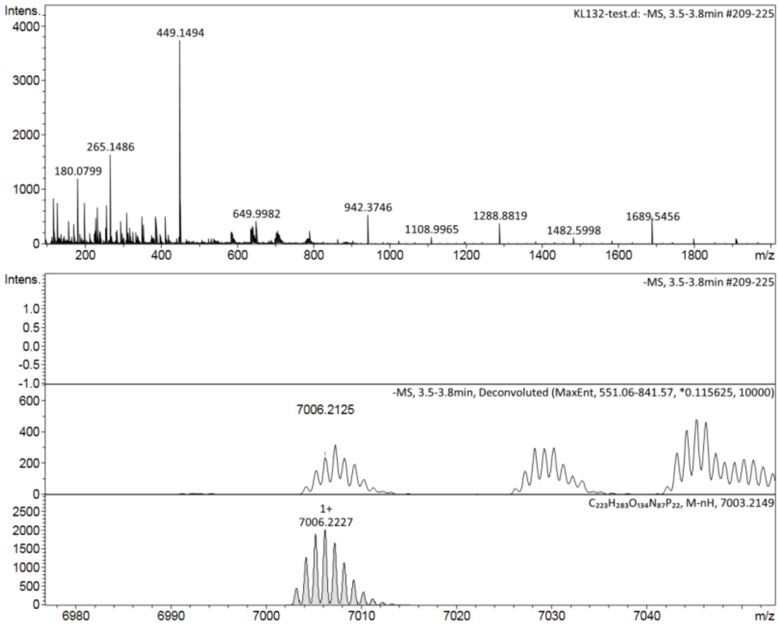

O[6]-ethyl-G:

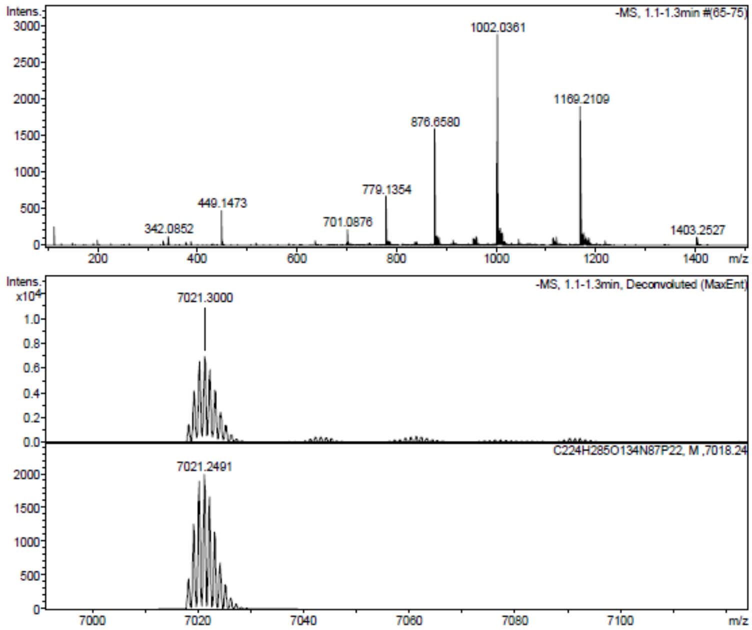

***O*[6]-methyl-G[gDNA]:**

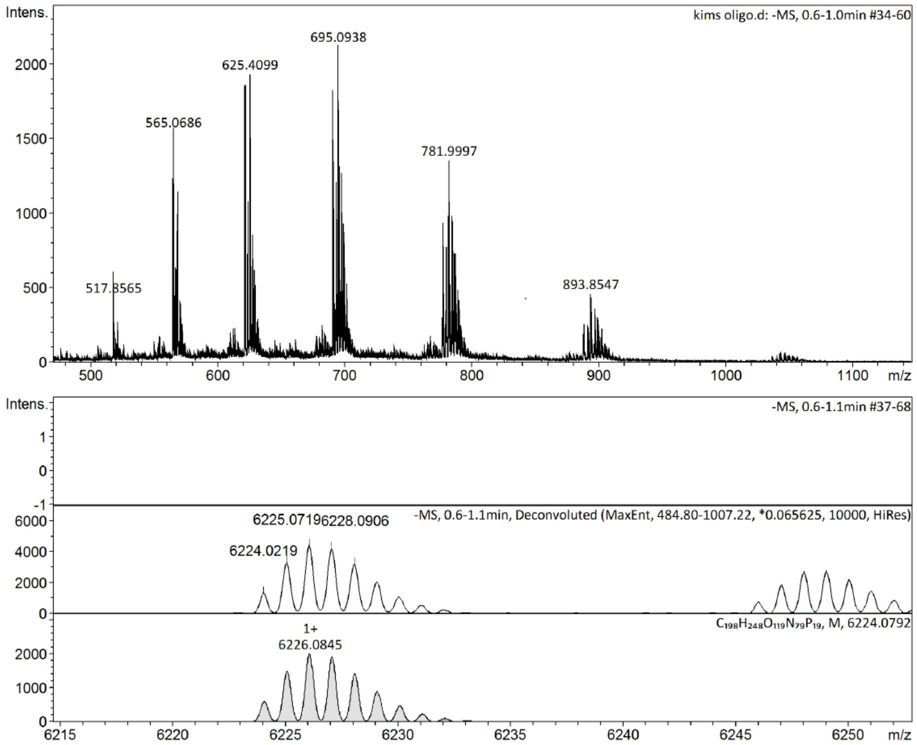

References:

[1] a) S. Obeid, N. Blatter, R. Kranaster, A. Schnur, K. Diederichs, W. Welte, A. Marx, *Embo J* **2010**, *29*, 1738-1747; b) K. Betz, D. A. Malyshev, T. Lavergne, W. Welte, K. Diederichs, T. J. Dwyer, P. Ordoukhanian, F. E. Romesberg, A. Marx, *Nat. Chem. Biol.* **2012**, *8*, 612-614; c) K. Bergen, K. Betz, W. Welte, K. Diederichs, A. Marx, *ChemBioChem* **2013**, *14*, 1058-1062.

[2] E. Nandanan, E. Camaioni, S. Y. Jang, Y. C. Kim, G. Cristalli, P. Herdewijn, J. A. Secrist, 3rd, K. N. Tiwari, A. Mohanram, T. K. Harden, J. L. Boyer, K. A. Jacobson, *J. Med. Chem.* **1999**, *42*, 1625-1638.

[3] F. Seela, P. Leonard, *Helv. Chim. Acta* **1997**, *80*, 1301-1318.

[4] G. S. Ti, B. L. Gaffney, R. A. Jones, *J. Am. Chem. Soc.* **1982**, *104*, 1316-1319.

[5] H. Lang, M. Gottlieb, M. Schwarz, S. Farkas, B. S. Schulz, F. Himmelsbach, R. Charubala, W. Pfleiderer, *Helv. Chim. Acta* **1999**, *82*, 2172-2185.

[6] a) J. Petruska, M. F. Goodman, M. S. Boosalis, L. C. Sowers, C. Cheong, I. Tinoco, Jr., *Proc. Natl. Acad. Sci. U. S. A.* **1988**, *85*, 6252-6256; b) M. S. Boosalis, J. Petruska, M. F. Goodman, *J. Biol. Chem.* **1987**, *262*, 14689-14696; c) S. Creighton, M. M. Huang, H. Cai, N. Arnheim, M. F. Goodman, *J. Biol. Chem.* **1992**, *267*, 2633-2639.
